# Secretomics reveals gelatinase substrates at the blood-brain barrier that are implicated in astroglial barrier function

**DOI:** 10.1126/sciadv.adg0686

**Published:** 2023-07-19

**Authors:** Miriam Burmeister, Annika Frauenstein, Martin Kahms, Laura Arends, Hanna Gerwien, Tushar Deshpande, Tanja Kuhlmann, Catharina C. Gross, Venu N. Naik, Heinz Wiendl, Juergen Klingauf, Felix Meissner, Lydia Sorokin

**Affiliations:** ^1^ Institute of Physiological Chemistry and Pathobiochemistry, University of Muenster, Münster, Germany.; ^2^ Cells-in-Motion Interfaculty Centre (CIMIC), University of Muenster, Münster, Germany.; ^3^ Max-Planck Institute for Biochemistry, Martinsried, Germany.; ^4^ Institute of Medical Physics and Biophysics, University of Muenster, Münster, Germany.; ^5^ Institute of Neuropathology, University Hospital Muenster, Münster, Germany.; ^6^ Neurology Department., University Clinic, University of Muenster, Münster, Germany.; ^7^ Brain and Mind Center,, Sydney, New South Wales, Australia.; ^8^ Institute of Innate Immunity, Department of Systems Immunology and Proteomics, Medical Faculty, University of Bonn, Bonn, Germany.

## Abstract

The gelatinases, matrix metalloproteinase 2 (MMP-2) and MMP-9, are key for leukocyte penetration of the brain parenchymal border in neuroinflammation and the functional integrity of this barrier; however, it is unclear which MMP substrates are involved. Using a tailored, sensitive, label-free mass spectrometry–based secretome approach, not previously applied to nonimmune cells, we identified 119 MMP-9 and 21 MMP-2 potential substrates at the cell surface of primary astrocytes, including known substrates (β-dystroglycan) and a broad spectrum of previously unknown MMP-dependent events involved in cell-cell and cell-matrix interactions. Using neuroinflammation as a model of assessing compromised astroglial barrier function, a selection of the potential MMP substrates were confirmed in vivo and verified in human samples, including vascular cell adhesion molecule–1 and neuronal cell adhesion molecule. We provide a unique resource of potential MMP-2/MMP-9 substrates specific for the astroglia barrier. Our data support a role for the gelatinases in the formation and maintenance of this barrier but also in astrocyte-neuron interactions.

## Teaser

Focal cleavage of selected cell surface proteins on astroglial cells modulate brain vessel barrier function.

## INTRODUCTION

The endothelium of cerebral blood vessels constitutes both a physical and a metabolic barrier (*1*, *2*); however, without interactions involving the subjacent astroglial layer, a fully functional blood-brain barrier (BBB) cannot form (*1*, *3*). In particular, studies of neuroinflammation have revealed that the endothelial and the astroglial layers represent molecularly and functionally distinct barriers to penetrating leukocytes (*4*). Using murine experimental autoimmune encephalomyelitis (EAE), a preclinical model for human multiple sclerosis , leukocytes were shown to first penetrate the endothelial barrier and accumulate in the perivascular space defined by the inner endothelial basement membrane (BM) and an outer parenchymal BM and associated astroglial layer, without the appearance of EAE symptoms (*5*, *6*). Disease symptoms develop only once leukocytes have penetrated the latter parenchyma border, highlighting its important contribution to the functional integrity of the BBB and its independence from the endothelial barrier. While considerable information is available on the molecular steps involved in leukocyte penetration of the endothelial barrier (*5*, *7*), little is known about the subsequent disease-limiting step.

The gelatinases, matrix metalloproteinase 2 (MMP-2) and MMP-9, are known to be activated only at sites of leukocyte penetration of the parenchymal border (*6*). In addition, in human multiple sclerosis , MMP-9 activity in the cerebrospinal fluid (CSF) is a marker of disease progression, and the use of injectable probes in molecular imaging has confirmed MMP-2/MMP-9 activity as an early marker of leukocyte infiltration into the brain parenchyma—to date, the only specific marker of an ongoing neuroinflammation (*8*). Both immune and brain-resident astrocytes and microglia are sources of gelatinases in EAE and in the absence of either enlarged perivascular cuffs or delayed disease onset result, due to less efficient penetration of the parenchymal border, while the absence of both sources ablates disease (*8*, *9*). MMP-2 is constitutively expressed in the noninflamed brain (*10*) and while both gelatinases are up-regulated during neuroinflammation, this is more pronounced for MMP-9 (*6*, *9*), suggesting that MMP-2 and MMP-9 have both beneficial and negative effects at the BBB. Hence, deciphering their substrate specificity at the parenchymal border will aid in the understanding of molecular processes that contribute to astroglial barrier function.

The only confirmed MMP-2/MMP-9 substrates at the astroglial border are β-dystroglycan, an integral component of an adhesion complex that anchors astrocyte endfeet to the parenchymal BM (*6*), and NOTCH1, the cleavage of which strongly promotes astrocyte expression of various chemokines, thereby promoting leukocyte migration across the astroglial barrier (*9*). The data are, therefore, sparse, and how the gelatinases affect barrier properties of parenchymal border remains unclear. This is largely due to limitations in the ability to detect small cleavage products in complex tissue or cell lysates such as the brain. Recent advancements in mass spectrometry (MS)–based proteomic workflows, instrumentation, and software solutions have, to some extent, overcome such problems and provided powerful tools for the discovery of proteolytic products (*11*, *12*). However, despite the development of more sensitive proteomic strategies to enrich protease substrates (*12*–*14*), comprehensive identification of explicit protease cleavage sites from primary cells or tissues remains challenging. We have previously described a sensitive label-free, MS-based approach to quantify low-picogram quantities of secreted proteins from primary immune cells (*15*, *16*). Using our knowledge on gelatinase sources at the parenchymal border in inflammatory conditions and their likely mode of altering barrier properties, we have now devised a method to specifically identify peptides derived from proteolytic cleavage of cell membrane–associated proteins. For this purpose, we identified and quantified annotated extracellular protein sequences separately from intracellular sequences with MaxQuant label-free quantitation (MaxLFQ) (*17*) rather than total protein as in standard secretomic experiments.

By comparing MMP-2/MMP-9–mediated cleavage products in conditioned media from wild-type (WT) astrocyte cultures with those lacking MMP-9 and/or MMP-2, we identified 119 MMP-9 and 21 MMP-2 potential substrates. These include known substrates (β-dystroglycan) (*6*, *9*) but predominantly molecules not previously identified as MMP substrates, including adhesion molecules, such as neuronal cell adhesion molecule (NrCAM), vascular cell adhesion molecule–1 (VCAM-1), cadherin-2, cadherin-4, and cadherin-11, and extracellular matrix (ECM) molecules, such as agrin, a known dystroglycan ligand (*6*, *9*). Our data suggest that inflammation-induced MMP-2/MMP-9 activity at the astroglial border selectively cleaves molecules involved in astrocyte-astrocyte and astrocyte-parenchymal BM interactions, likely to compromise not only barrier function but also astrocyte-neuron interactions. We confirm VCAM-1 as an inflammation-induced MMP-9 substrate on astrocytes and glial cells, the cleavage of which inhibits T cell binding at the astroglial border and differentiation to the pathogenic T helper cell 17 (T_H_17) phenotype, thus acting as a negative regulator of leukocyte penetration of the BBB. Our study provides a unique resource for potential gelatinase substrates at the astroglial barrier and provides new mechanistic insights into proteolytic events occurring specifically at the parenchymal border when barrier function is impaired.

## RESULTS

### Astrocytes as sources of gelatinases at the parenchymal border

Both MMP-9 mRNA (*Mmp9)* and *Mmp2* are expressed by astrocytes and glia at the parenchymal border (*6*, *9*). Therefore, to investigate MMP-2 and MMP-9 substrates at this border, we used astroglial cultures from WT, *Mmp2^−/−^, Mmp9^−/−^,* and *Mmp2^−/−^/Mmp9^−/−^* double knockout (DKO) mice. As MS detection of protein substrates released into the extracellular space is challenging in the presence of highly abundant serum proteins, untreated astroglial cultures were grown overnight in serum-free, nutrient-rich Freestyle medium before collection of conditioned media for analysis. We evaluated the expression of MMP-2 and MMP-9 under these conditions using gelatin gel zymography, revealing less MMP activity than in serum-containing medium, due to the presence of activated MMPs in serum (*6*), but detectable expression of both pro- and activated MMP-2 and MMP-9 (fig. S1A). The cellular composition of the cultures under serum-free conditions as revealed by flow cytometry for CD11b^+^ microglia and glutamate aspartate transporter (GLAST)^+^ astrocytes were similar and consisted of 90% GLAST^+^ and 10% CD11b^+^ cells (fig. S1B); they are, therefore, referred to as astrocyte cultures. Cell viability, determined by flow cytometry, was >88% and did not differ between the cultures (fig. S1C).

As an additional control, WT astrocytes were cultured overnight with and without treatment with the chemical inhibitor, (S)-3-methyl-2-{4-[3-(5-methylthiophen-2-yl)-1,2,4-oxadiazol-5-yl]phenylsulfonamido}butanoic acid (MOBSAM), at a concentration that inhibits both MMP-2 and MMP-9 activation (*18*). Flow cytometry confirmed that MOBSAM did not adversely affect cell viability (fig. S1D).

### Tailored secretomics to identify protease substrates at astrocyte cell surface

We performed secretome analyses from conditioned media of WT; *Mmp2^−/−^, Mmp9^−/−^,* and *Mmp2^−/−^/Mmp9^−/−^* DKO astrocytes; and WT cells treated with MOBSAM (fig. S2, A and B). Proteins in the conditioned media were digested with trypsin and endoproteinase LysC, and the resulting peptide mixtures were analyzed by liquid chromatography coupled to MS (LC-MS/MS). Raw MS data were analyzed in the MaxQuant environment (*19*). As gelatinases act mainly extracellularly, we predicted that the abundance of extracellularly annotated peptides would be lower in secretomes of MMP knockouts (KOs), as elimination of MMPs would result in less cleavage of substrates and fewer peptides released into the supernatant. By contrast, peptides derived from intracellular protein domains would not change. Accordingly, we classified identified peptides as intra- or extracellular.

**Fig. 1.**
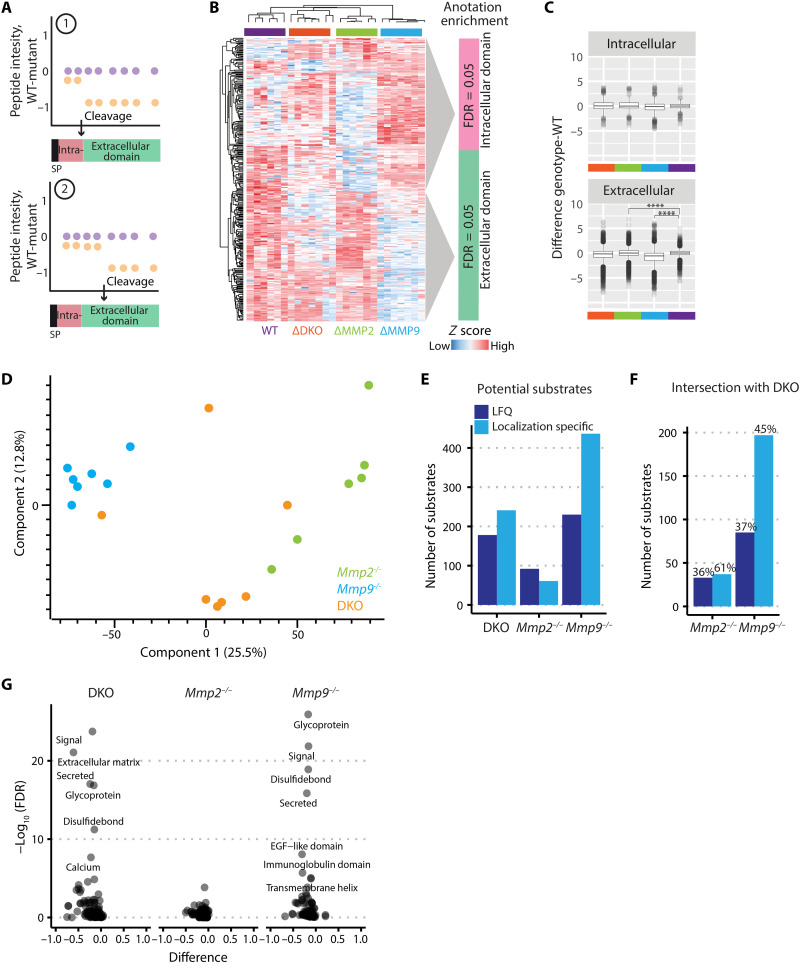
Secretome analyses of proteins shed from astrocytes. (**A**) Schematic cleavage patterns by MMPs cleaving either at the border between intra- and extracellular protein domains (1) or within extracellular domains (2). (**B**) Hierarchical clustering (Pearson correlation) of statistically significantly regulated peptides of potential MMP-9 substrates (415 peptides corresponding to 243 proteins), showing differential enrichment of intracellular versus extracellular domains in the two major clusters. FDR, false discovery rate. (**C**) Differences between genotypes of intra- or extracellular peptides from potential MMP-9 substrates. Each boxplot represents six independent measurements. Student’s *t* test; ****P < 0.0001. (**D**) PCA of the extracellular and not specified protein domains of proteins with membrane annotation (9275 peptides corresponding to 1328 proteins) from *Mmp2^−/−^*, *Mmp9^−/−^,* and DKO-conditioned media normalized to WT. Component 1 accounts for 25.5% of the data variation. (**E**) Number of statistically significant released proteins (Student’s *t* test, *P* value of < 0.03) identified by LFQ (dark blue) versus localization-specific quantification (light blue). (**F**) Intersection of statistically significant released proteins identified in *Mmp2^−/−^* and DKO, and *Mmp9^−/−^* and DKO samples by LFQ (dark blue) versus localization-specific quantification (light blue). The percentage refers to the fraction of intersecting substrates compared to total number of substrates identified for each genotype and quantification method. (**G**) 1D annotation enrichment of UniProt keywords for the statistically significant released proteins identified for *Mmp2^−/−^*, *Mmp9^−/−^*, and DKO in comparison to WT, respectively.

We next assessed two modes of generating extracellular peptides: (i) cleavage at the interface between extra- and intracellular protein domains or, alternatively, (ii) cleavage anywhere within the extracellular domain (Fig. 1A). The peptides in these two categories that showed statistically significant differences in abundance between WT versus *Mmp9^−/−^*, *Mmp2^−/−^*, or DKO secretomes were subjected to hierarchical clustering; an example for WT versus *Mmp9^−/−^* is shown in Fig. 1B where 415 peptides were detected, corresponding to 243 proteins (Fig. 1B). As predicted, extracellular peptides were significantly reduced in *Mmp9^−/−^, Mmp2^−/−^*, and DKO samples compared to WT, while intracellular peptides were not different (Fig. 1C). Corresponding quantifications of intra- or extracellular peptides in *Mmp2^−/−^* and DKO samples are shown in fig. S3 (A and B). A principal components analysis (PCA) with extracellular peptide intensities showed distinct clusters for *Mmp2^−/−^, Mmp9^−/−^*, and DKO samples, with the greatest difference between *Mmp2^−/−^* and *Mmp9^−/−^* samples along component 1, representing 25% of the data variation (Fig. 1D). Less data variance was apparent when all peptides (22.7%) or intracellular peptides (21.1%) were analyzed (fig. S3, C and D). We identified more outliers for *Mmp9^−/−^* and DKO samples using our subcellular localization-specific quantification strategy compared to conventional label-free quantification (LFQ)–based secretome analyses (Fig. 1E), and intersecting substrates identified in single KO and DKO samples were increased (Fig. 1F), suggesting more precise analyses. For example, the extracellular peptides of the previously described MMP substrate, β-dystroglycan (DAG1), were significantly regulated with our analysis strategy (fig. S3E), whereas standard LFQ secretome analyses did not reveal statistically significant regulation (fig. S3F).

We used one-dimensional (1D) annotation enrichment to bioinformatically determine global differences among the potential MMP-2 and MMP-9 substrates compared to WT. This identified proteins with the UniProt annotations “ECM” and “secreted” among the most relevant (Fig. 1G), highlighting that the identified substrates are derived from the extracellular protein pool, where MMPs are active.

### Identification and quantification of MMP-2 and MMP-9 substrates

**Fig. 2.**
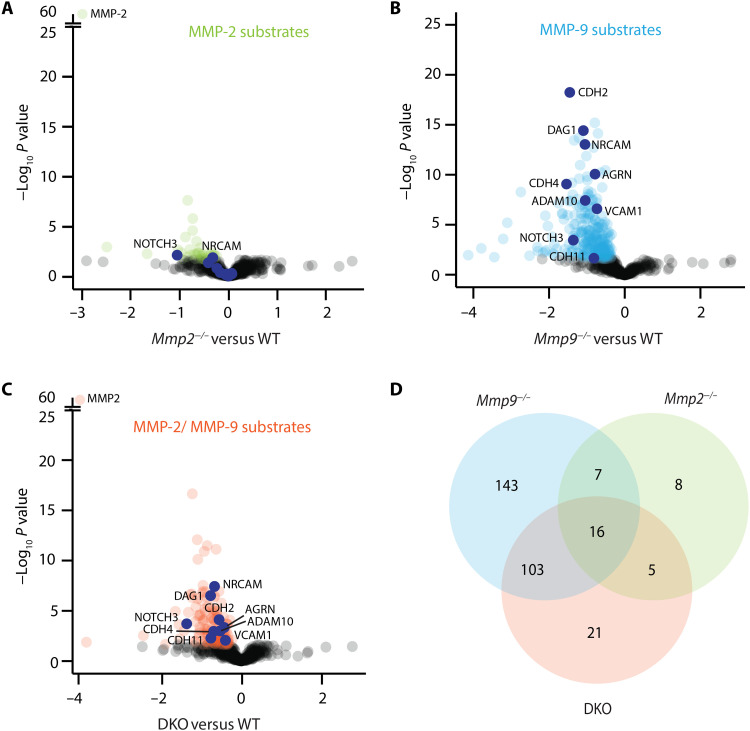
Differentially shed proteins upon genetic MMP ablation. Volcano plots showing differentially changing extracellular and not specified protein domains detected in the different MMP-conditioned media. Pairwise comparisons to define (**A**) MMP-2–, (**B**) MMP-9–, and (**C**) DKO-specific substrates. *P* values (−log_10_) are plotted against the difference (log_2_) between the genotypes (Student’s *t* test). The *y* axis of (A) and (C) were split to allow visual comparison between genotypes. Statistically significant released proteins (*P* < 0.03) by MMPs are highlighted in green (MMP-2), blue (MMP-9), or orange (MMP-2 and MMP-9). Proteins that were further investigated are marked in dark blue. (**D**) Venn diagram showing extracellular domains that were down-regulated in *Mmp2^−/−^*, *Mmp9^−/−^*, and DKO samples compared to WT (i.e., substrates) and intersections of common substrates identified.

To identify MMP-9 and MMP-2 substrates, we calculated differences in the extracellular peptides between KOs and WT secretomes and assessed the significance of changes by two-sided *t* tests. The results are visualized in volcano plots, where the *P* values are plotted against the difference between the genotypes. Extracellular peptides that were less abundant in the supernatant of the KO secretomes were considered potential substrates. Our analyses revealed the highest number of substrates for MMP-9 (269), while MMP-2 (36) showed less proteolytic activity under the conditions used; many substrates were confirmed in DKO samples (145) (Fig. 2, A to C). Treatment with MOBSAM led to the identification of 177 potential MMP substrates (fig. S4A). Although the identification of intracellular peptides is challenging in the case of a small intracellular protein domain or low proteolytic accessibility due to membrane association, we detected intracellular domains of 90 proteins with our approach, which remained mainly constant across all genotypes (fig. S4B). In addition, we controlled for differences in protein expression levels in KO cells by analyzing the cell lysates (i.e., full proteomes) of *Mmp2^−/−^, Mmp9^−/−^*, and WT samples using the same bioinformatical pipeline, e.g., splitting the peptides into their subcellular localizations before statistical analysis (fig. S4C). Only 23 substrates also showed a statistically significant change at the total proteome level and were excluded from further analysis.

To explore our proteomic data and to visualize potential MMP-2 and MMP-9 substrates easily, we developed a web-based interface (http://gelatinasesubstrates.immprot.cloud) (Shiny app, R). The interactive volcano plots and interactive tables allow direct comparison of substrates identified in the different genotypes and in chemical inhibition experiments, including changes in the full proteome and in intracellular domains. In addition, the Shiny app shows the differential peptide intensity distribution for extracellular and nonspecified domains of the potential substrates. Where applicable, cytoplasmic domains are also shown as a control.

Comparison of the significantly less abundant peptides in the single KOs and DKO secretomes compared to WT samples revealed 119 molecules common to *Mmp9^−/−^* and DKO samples and 21 molecules common to *Mmp2^−/−^* and DKO samples, of which 16 were common to all three samples (Fig. 2D and fig. S2B). Of the 16 common molecules, approximately 37.5% were additionally confirmed by analysis of the MOBSAM-treated samples, which is likely to be related to the efficiency of inhibition under the conditions used. Substrates that were specific for *Mmp9^−/−^*, *Mmp2^−/−^*, or DKO samples alone may reflect peptides resulting from the other proteolytic processes or those that have not reached statistical significance. The suitability of our secretome strategy was verified by the identification of β-dystroglycan (DAG1) as a highly statistically significant substrate for MMP-9 (Fig. 2). We had previously identified β-dystroglycan as a MMP-2 and MMP-9 substrate associated with BBB breakdown in neuroinflammation (*6*). Our results here do not mean that β-dystroglycan is not cleaved by MMP-2 but that MMP-9 is more efficient in doing so.

Potential MMP-2 substrates that were confirmed in DKO and in some cases MOBSAM-treated samples were largely associated with early endosomes, mitochondrial, or Golgi/endoplasmic reticulum (ER) membranes and, hence, may reflect proteins stemming from exosomes or vesicles secreted into the media by the cells. In addition, MRC1 (mannose receptor C type 1), GLYCAM1 (glycosylation-dependent cell adhesion molecule-1), IGF2R (insulin-like growth factor 2 receptor) (NOTCH3, PDGFRB (platelet-derived growth factor receptor-β) , and NrCAM, all known to be expressed by astrocytes (*20*–*22*), were identified as to date unknown potential MMP-2 substrates. By contrast, potential MMP-9 substrates, which were confirmed in DKO samples, were abundant and associated mainly with cell-cell [cadherin-2, cadherin-4, cadherin-11, and cadherin-19/cadherin-25 (DCHS1), VCAM-1, NrCAM, NCAM1 (neural cell adhesion molecule 1), MXRA8 (limitrin), PTPRK (protein tyrosine phosphatase receptor type K), and NOTCH3] and cell-matrix adhesion [DAG1, MCAM (melanoma cell adhesion molecule), PTPRS (protein tyrosine phosphatase receptor type S), AGRN (agrin), COLVIa2 (collagen type VI a2 chain), GPC1, and GPC4 (glypican 1 and 4)], as well as exosomes/vesicles as for MMP-2, and catalytic activity (MMP14, ADAM10, ADAM12, and MMP19). Among the 16 common potential MMP-2 and MMP-9 substrates, several cell surface molecules were highly significant, including NrCAM and NOTCH3, which are associated with astrocyte function (Fig. 2) (*22*, *23*). We had previously identified NOTCH1 (*9*) as an MMP-2 and MMP-9 substrate that is structurally very similar to NOTCH3, thus further strengthening the suitability of our approach.

### Experimental validation of MMP substrates

Because MMP-9 activity is highly up-regulated at the astroglial barrier during inflammation where the barrier function is compromised when leukocytes penetrate (*9*), we focused on to date unknown MMP-9 targets, including those shared with MMP-2, which were confirmed in DKO and in most cases MOBSAM samples and, additionally, had a cell adhesion, ECM binding, or cell junction annotation and were not associated with exosomes/vesicles (g:Profiler) (fig. S5, A and B). In addition to NrCAM and NOTCH3, the highly statistically significant MMP-9 substrates identified were DAG1 (dystroglycan), as expected, plus several cadherins—cadherin-2 (also known as N-cadherin), cadherin-4, cadherin-19/cadherin-25 (DCHS1), and cadherin-11—and cadherin-associated molecules [CLSTN1 (calsyntenin-1), PTPRK, PTPRS, and AP2A2 (AP-2 complex subunit alpha-2)], plus the adhesion molecules NCAM1, VCAM-1, and MXRA8 and the extracellular matrix molecules AGRN and COL6A2 and the cell surface proteoglycansGPC1 andGPC4). All molecules were confirmed to be expressed by astrocytes based on published single cell RNA sequencing (scRNASeq) or other data (*9*, *24*) but several could not be further investigated due to lack of specific tools. On the basis of our knowledge of the astroglial border and MMP activity at this site (*6*, *8*, *9*) and published information on cleavage by gelatinases (*12*, *13*), we selected eight molecules (cadherin-2, cadherin-4, cadherin-11, VCAM-1, NrCAM, NOTCH3, agrin, and ADAM10) for further investigation (Fig. 2B).

### Quantitative PCR and immunofluorescence staining to confirm expression of substrates by astrocytes

**Fig. 3.**
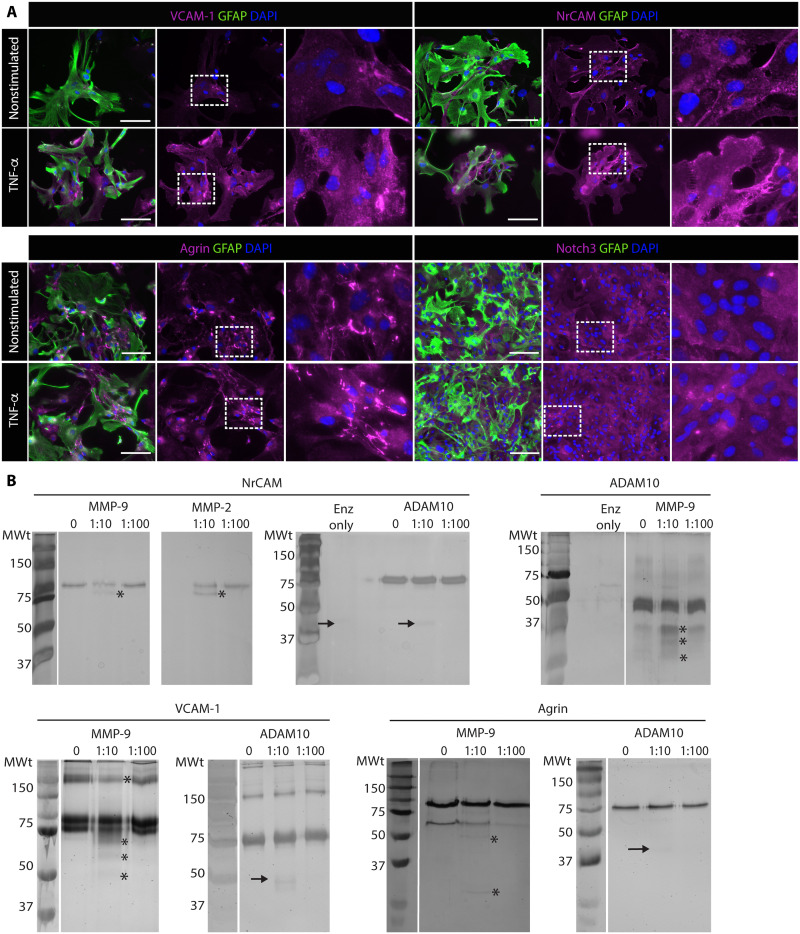
Expression and cleavage of selected gelatinase substrates in TNF- α and unstimulated WT astrocyte cultures. (**A**) Immunofluorescence staining for MMP substrates, VCAM-1, NrCAM, agrin, NOTCH3, together with GFAP to mark astrocytes and DAPI; boxed areas are shown to the right at higher magnifications. Scale bars, 100 μm. (**B**) Silver-stained gels showing cleavage products of gelatinase substrates after overnight incubation without (0) or with 1:10 or 1:100 ratios of MMP-9:substrate or ADAM10:substrate. Arrows mark the positions of ADAM10 in samples. Asterisks mark specific cleavage products. Data are representative of two to three experiments.

Gene expression of these eight molecules in WT astrocytes was confirmed using polymerase chain reaction (PCR) (fig. S6A); additionally, where possible, protein expression was verified using immunofluorescence staining (Fig. 3A and fig. S7A). Similar results were obtained in DKO astrocytes (fig. S6B, immunofluorescence not shown), indicating that loss of gelatinase expression does not affect the synthesis of these substrates.

### In vitro cleavage and SDS-PAGE to verify MMP substrates

Analyses of cell lysates from WT versus DKO astrocytes using Western blots in most cases did not reveal clear differences in the sizes of any of the eight substrates or detect fragments, probably due to antibody specificities and/or low protein amounts and/or the small size of cleavage products. In vitro cleavage assays were, therefore, used where full-length recombinant proteins or extracellular domains were available (NrCAM, VCAM-1, N-cadherin, cadherin-4, cadherin-11, agrin, and ADAM10). Activated MMP-9 was incubated overnight at 37°C with a 10- or 100-fold excess of substrate, and cleaved products were identified by silver staining after separation of proteins by SDS–polyacrylamide gel electrophoresis (SDS-PAGE) (*12*). All substrates tested showed some degree of cleavage with the higher MMP-9 concentration (Fig. 3B and fig. S7B). As NrCAM was identified as a common MMP-2 and MMP-9 substrate, in vitro cleavage of this molecule by MMP-2 was also tested, revealing the same cleavage product as that obtained with MMP-9 (Fig. 3B).

### ADAM10 and MMP-2/MMP-9 show distinct cleavage patterns

ADAM10 is a transmembrane protein with both adhesion and protease domains that can cleave both NOTCH1 and NOTCH3 (*25*) and has been reported to cleave N-cadherin (*26*) and NrCAM (*27*). This raised the question whether some of the differences detected between MMP-deficient and WT-conditioned media could result from indirect effects, i.e., MMP-2– or MMP-9–mediated release of cell surface ADAM10. We, therefore, tested whether activated ADAM10 could cleave the identified substrates. While N-cadherin and cadherin-11 were cleaved by ADAM10, the other substrates including VCAM-1, NrCAM, agrin, and cadherin-4 were not (Fig. 3B and fig. S7B). The predicted ADAM10 cleavage site in NrCAM (*28*) is not present in the recombinant protein used in the in vitro cleavage assays, which consists only of the immunoglobulin (Ig)–like domains (AS30-630), confirming that the MMP-9 and ADAM10 cleavage sites are distinct from each other. In addition, the cleavage products obtained with MMP-9 and ADAM10 differed for N-cadherin and cadherin-11, suggesting that both proteases can cleave these substrates (fig. S7B) but at different sites. ADAM10 mRNA was extensively expressed in the central nervous system (CNS) parenchyma surround cuffs, probably in neurons as previously reported (*29*), but also by astrocytes as shown by RNAscope of glial fibrillary acidic protein (GFAP)^+^ astrocytes in culture (fig. S8, A and B). Hence, cell surface–activated ADAM10 may contribute to cleavage of NOTCH, which we have previously reported to be an MMP-9 substrate on astrocytes (*9*), and its shedding from the astrocyte surface is probably mediated by MMP-9, as also suggested by the in vitro cleavage assay. Such shedding is likely to stop the proteolytic action of ADAM10 on cell surface proteins.

### Confirmation of MMP substrates under inflammatory conditions

To investigate whether expression levels were altered under inflammatory conditions, PCR was performed on tumor necrosis factor–α (TNF-α)–treated WT astrocyte cultures, revealing increased expression not only of VCAM-1, as shown previously (*30*), but also of agrin and, to a lesser extent, cadherin-4 compared to untreated cultures (fig. S6A); these data were confirmed by immunofluorescence staining (Fig. 3A and fig. S7A). Immunofluorescence staining for NrCAM and cadherin-11 was also enhanced under TNF-α treatment (Fig. 3A and fig. S7A).

### Validation of MMP substrates in vivo in EAE

All molecules were detected by immunofluorescence staining at the parenchymal border in naïve and EAE brains, confirming in vivo relevance, but not all were altered at sites of leukocyte penetration of the parenchymal border where MMP activity occurs (fig. S9). While the absence of changes in expression under TNF-α stimulation and/or in in vivo and in vitro immunofluorescence staining patterns does not exclude involvement in parenchymal border barrier function, we focused on molecules showing the clearest changes in EAE and/or representing previously unknown gelatinase substrates, i.e., NrCAM and VCAM-1.

**Fig. 4.**
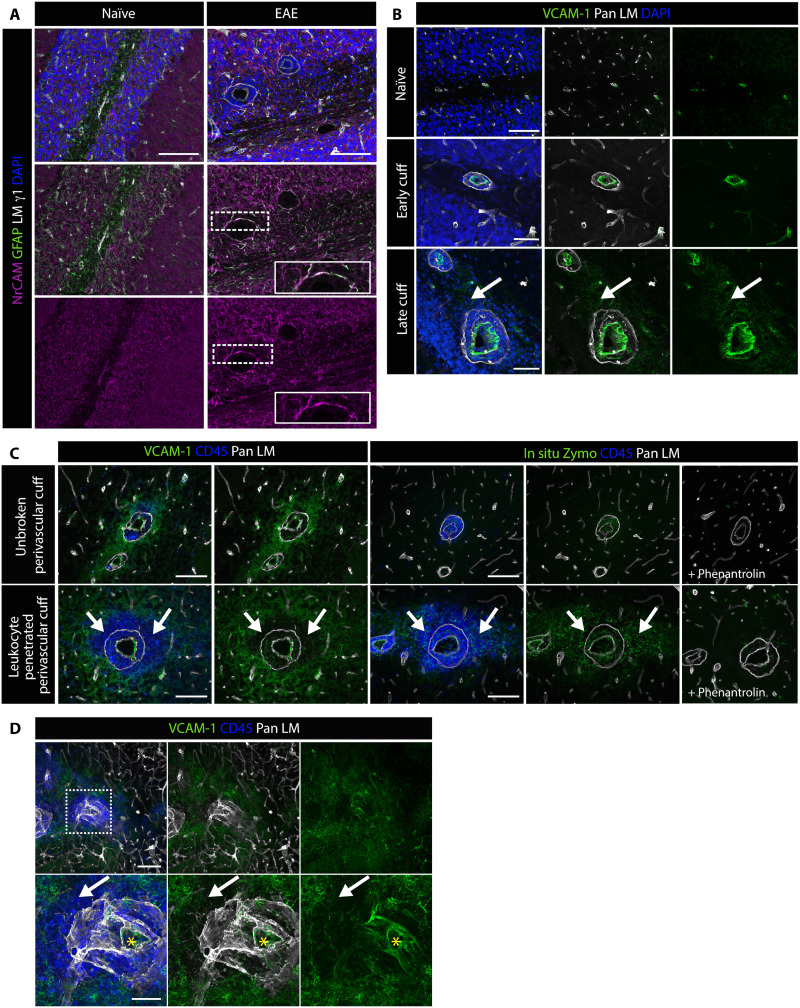
In vivo expression of MMP substrates, NrCAM and VCAM-1, in noninflamed (naïve) and EAE brains. WT brain sections were immunofluorescently stained for (**A**) GFAP to mark astrocytes, anti–laminin-γ1 chain antibody to mark BMs and perivascular cuffs, and NrCAM; DAPI marks all nuclei; scale bars, 100 μm; areas marked by the dotted lines are shown at higher magnifications in boxed areas. (**B**) Immunofluorescence staining for VCAM-1 and pan-laminin (Pan LM) in naïve and in early- and late-stage EAE brains; arrow marks VCAM-1 in CNS parenchyma at late-stage EAE; DAPI marks nuclei; scale bars, 100 μm (naïve) and 50 μm (early/late cuff). (**C** and **D**) Triple immunofluorescence staining for CD45, Pan LM, and VCAM-1 shows up-regulation of VCAM-1 around inflammatory cuffs, and its loss at this site where CD45 infiltration occurs (arrows), which correlates with sites of gelatinase activity as shown by in situ zymography (C, bottom) performed on consecutive sections; images to the far right in (C) show in situ hybridizations performed in the presence of the MMP inhibitor, 1,10-phenantrolin. Boxed area in (D) is shown at higher magnification in bottom panels; yellow asterisks mark vessel lumen. Scale bars, 100 μm (C) and 50 μm (D).

NrCAM staining colocalized with GFAP^+^ astrocytes surrounding blood vessels in the naïve brain and strongly enhanced surrounding perivascular cuffs in EAE brains but was not altered at sites of leukocyte penetration of the parenchymal border (Fig. 4A). Similar results were obtained with single KO samples, consistent with cleavage of NrCAM by both gelatinases (fig. S10A). While VCAM-1 has been previously shown to be expressed by astrocytes in vivo (*30*), we observed temporal-spatial differences with cuff development (Fig. 4B). As previously reported, VCAM-1 staining of endothelium increased at early stages of cuff development (*31*) with enhanced and broader staining of the surrounding tissue at late-stage cuffs but only when immune cells were confined within the parenchymal border (Fig. 4, B and C). By contrast, at sites where leukocytes had penetrated the parenchymal border and infiltrated into the CNS parenchyma, VCAM-1 staining was reduced or completely lost (Fig. 4, C and D). The same pattern of results was observed in *Mmp2^−/−^* samples, but there was no loss of VCAM-1 staining around leukocyte-penetrated cuffs in *Mmp9^−/−^* samples (fig. S10, B and C), consistent with VCAM-1 being mainly an MMP-9 substrate. DKO mice do not develop EAE and showed the same pattern of VCAM-1 as WT naïve samples (fig. S10C). While these results may indicate complete loss of VCAM-1 at sites of leukocyte infiltration, they could also arise from loss of the epitope recognized by the VCAM-1 monoclonal antibody or a conformational change resulting from MMP cleavage. As ADAM17 has been reported to cleave VCAM-1, we also investigated the expression of ADAM17 in EAE brains, revealing no correlation with sites of leukocyte extravasation of the astroglia border.

In situ gel zymography confirmed the expression of activated gelatinases at sites of VCAM-1 loss and leukocyte accumulation (Fig. 4C). RNAscope performed on EAE sections confirmed expression of *Vcam1* in GFAP^+^ astrocytes surrounding cuffs (in addition to the already reported expression by endothelium) (fig. S11). The restricted *Vcam1* signal in the CNS parenchyma suggests that it is not expressed by neurons, which is supported by published imaging and recent scRNASeq data (*32*).

To confirm NrCAM or VCAM-1 cleavage in EAE brains, we performed immunoblotting of cerebellum extracts (where EAE cuffs predominate) and of CSF that would contain only shed/soluble proteins. As CSF samples from mice are <10 μl and protein levels are extremely low, only dot bots were possible on these samples. Although cleavage fragments could not be detected, Western blots of cerebellum extracts revealed less full-length VCAM-1 in WT and *Mmp2^−/−^* compared to *Mmp9^−/−^* samples in (fig. S12A), while dot blots of CSF revealed the opposite pattern, i.e., elevated sVCAM-1 signal in WT compared to *Mmp9^−/−^* samples (fig. S12B). As DKO do not develop EAE symptoms, these samples were compared to WT naïve brain samples. Both in Western blots of WT and DKO cerebellum extracts and in dot blots of CSF samples, VCAM-1 levels were not detectable as in the absence of inflammation it is lowly expressed, as expected from immunofluorescence staining of noninflamed brains (Fig. 4B). Western blots for NrCAM in cerebellum extracts revealed robust signals in all samples, due to its expression in neurons as well as astrocytes (fig. S12C) (*23*). However, in CSF samples from EAE mice, NrCAM levels were lower in *Mmp9^−/−^* samples compared to WT samples, consistent with its cleavage by both MMP-2 and MMP-9; as expected in the absence of inflammation and/or under conditions of low MMP activity, as occurs in DKO and naïve WT samples, the soluble NrCAM signal was low (fig. S12D).

### Functional significance of soluble VCAM-1

**Fig. 5.**
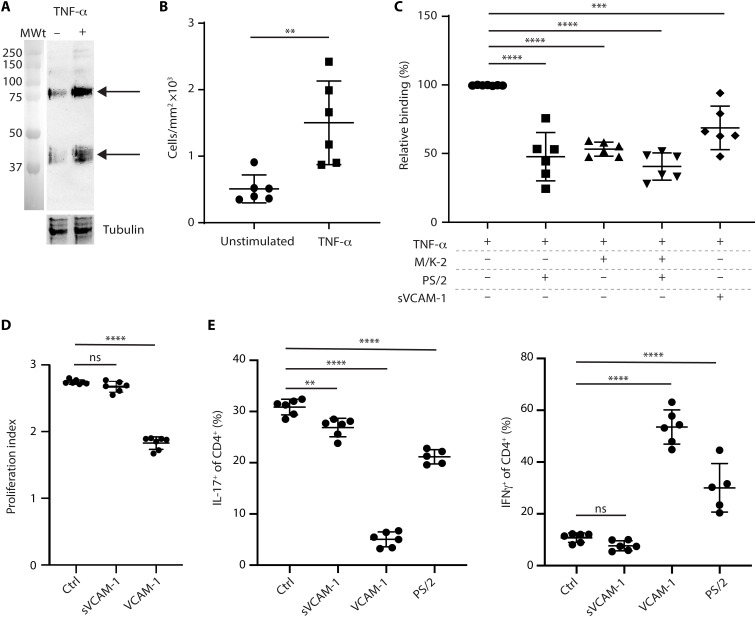
Function of cell membrane and soluble VCAM-1 on astrocyte–T cell interactions. (**A**) Western blot for VCAM-1 and tubulin control in cell lysates from unstimulated or TNF-α–stimulated WT astrocytes; arrows mark the two VCAM-1 isoforms (*35*). (**B**) Adhesion of primary encephalitogenic CD4^+^ T cells to unstimulated or TNF-α–stimulated WT astrocytes; data are means ± SD of six experiments performed with separate astrocyte preparations with three replicates per experiment; statistical analysis was Mann-Whitney; **P < 0.01. (**C**) Adhesion of primary encephalitogenic CD4^+^ T cells to TNF-α–stimulated WT astrocytes in the presence of blocking antibodies to VCAM-1 (M/K-2) or integrin α4β1 (PS/2) or soluble VCAM-1 (sVCAM-1); data are percentage of binding in the absence of blocking factors and are means ± SD of six to seven experiments performed with separate astrocyte preparations with three replicates per experiment; statistical analyses were ordinary one-way ANOVA; ***P < 0.001 and ****P < 0.0001. (**D**) In vitro proliferation of CD4^+^ encephalitogenic T cells in the presence or absence (ctrl) of soluble VCAM-1 (sVCAM-1) or of cells plated on VCAM-1; data are means ± SD of two experiments with three to four replicates per experiment. (**E**) In vitro T_H_1 and T_H_17 differentiation assays performed with encephalitogenic T cells in the presence or absence (ctrl) of sVCAM-1 or cells plated on VCAM-1 or PS/2; data are representative of three to four experiments with three to six replicates per experiment; statistical analyses in (D) and (E) are Welch ANOVA for multiple comparisons; **P < 0.01 and ****P < 0.0001.

As VCAM-1 supports encephalitogenic T cell adhesion and transmigration of the endothelial barrier (*33*), we investigated whether a similar mechanism exists at the astroglial barrier. TNF-α treatment of astrocytes enhanced expression of the two known VCAM-1 isoforms (*34*), as shown by Western blot (Fig. 5A), and promoted binding of primary encephalitogenic CD4^+^ T cells compared to untreated cells (Fig. 5B). Binding was reduced by preincubation of T cells with blocking antibodies to VCAM-1 (M/K-2), its receptor integrin α4β1 (*35*) (PS/2), or by preincubation with recombinant soluble VCAM-1 (Fig. 5C). This indicates integrin α4β1-mediated binding of encephalitogenic T cells to VCAM-1 on the surface of astrocytes and that soluble VCAM-1 can displace this binding.

To define the function of these interactions, we investigated whether in vitro proliferation of encephalitogenic [myelin oligodendrocyte glycoprotein (MOG)#x2013;specific] CD4 T cells is affected by immobilized VCAM-1 (as on the surface of astrocytes) or soluble VCAM-1 (as occurs upon MMP cleavage), revealing significantly lower proliferation of cells bound to VCAM-1, while soluble VCAM-1 had no effect (Fig. 5D). Furthermore, CD4^+^ T cells bound to plated VCAM-1 showed reduced T_H_17 and increased T_H_1 differentiation, effects that were mimicked to some extent when cells were cultured on plated anti-integrin α4β1 antibody (Fig. 5E). By contrast, soluble VCAM-1 had slight inhibitory effects on both T_H_17 and T_H_1 differentiation (Fig. 5E). This suggests that binding of infiltrating CD4^+^ T cells with VCAM-1 on the astrocyte cell surface at the parenchymal border limits T cell proliferation and differentiation toward the pathological T_H_17 phenotype and that these effects are ablated upon VCAM-1 cleavage by the gelatinases.

### NrCAM cleavage modulates ratios of excitatory/inhibitory synapses

NrCAM has been recently shown to be enriched at astrocyte-neuron junctions where astrocyte NrCAM interactions with neuronal NrCAM promote inhibitory GABAergic synapse formation between neurons (*23*). In addition, NrCAM has been reported to be cleaved by MMP-9; however, the functional significance of this cleavage is not clear (*36*) or whether such cleavage occurs in the CNS. We therefore investigated inhibitory (GABAergic) and excitatory synapse (glutamatergic) formation in vitro in astrocyte/neuron cocultures from WT, *Mmp2^−/−^, Mmp9^−/−^*, or DKO pups using immunofluorescent staining and super-resolution confocal microscopy (*23*).

**Fig. 6.**
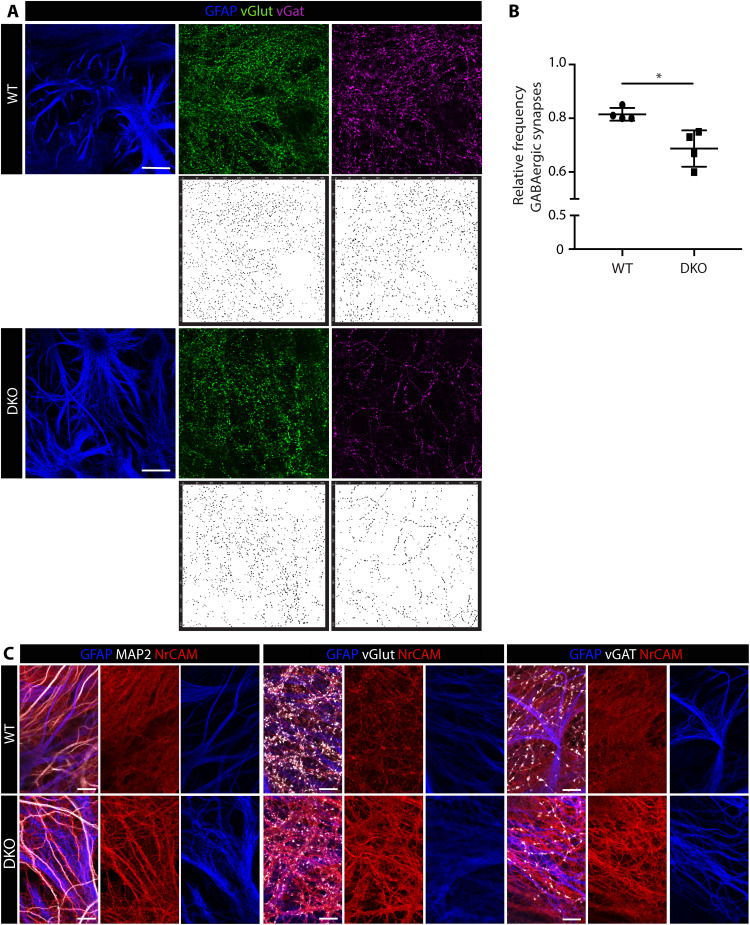
NrCAM function on astrocytes. (**A**) Immunofluorescence staining of WT and DKO astrocyte-neuronal cocultures for vGlut to mark excitatory synapses and vGAT to mark inhibitory synapses, plus GFAP to mark astrocytes. Wavelet transformations of synapse stainings are shown in bottom panels; scale bars, 20 μm. (**B**) Corresponding statistical analysis of four experiments with separate culture preparations; data are expressed as relative frequency of GABAergic compared to glutamatergic synapses. Data are means ± SD with two replicates and seven to eight regions analyzed per experiment with each region comprising around 500 to 1000 synapses. Statistical analysis was Student’s *t* test; *P < 0.05. (**C**) Immunofluorescence staining of NrCAM, GFAP, and either MAP2 to mark neurons, vGlut, or vGAT in WT and DKO astrocyte-neuronal cocultures; scale bars, 10 μm.

Whether MMP-2 and MMP-9 can be secreted by neurons was first tested by gelatin gel zymography of the conditioned medium from WT neurons cocultured with WT astrocytes, revealing a predominance of MMP-2 secretion as in astrocyte cultures alone (figs. S13A and S1A), as reported previously (*9*), suggesting little or no MMP contribution by the neurons. Cocultures of *Mmp2^−/−^*, *Mmp9^−^*, and DKO astrocytes and neurons similarly showed that astrocytes were the main source of gelatinases (fig. S13A). Coculture of DKO neurons with astrocytes resulted in a statistically significant decrease in the relative frequency of inhibitory GABAergic synapses compared to WT cocultures (Fig. 6, A and B) but no differences in *Mmp2^−/−^* or *Mmp9^−/−^* cocultures compared to WT controls. This confirms NrCAM as a common substrate for both gelatinases and indicates that MMP-2 and MMP-9 can, to some degree, compensate for each other (fig. S13B). *Mmp2^−/−^* cultures resembled the DKO phenotype, showing reduced, albeit not significant, GABAergic synapses between neurons, which is explained by higher levels of MMP-2 secreted by astrocytes compared to MMP-9 (fig. S13A). Immunofluorescence staining of the cocultures for NrCAM, GFAP, and either microtubule-associated protein 2 (MAP2) to mark neurons, vesicular glutamate transporter (vGlut), or vesicular GABA transporter (vGAT) confirmed more intense staining for NrCAM not only on DKO astrocytes but also on neurons, suggesting that astrocyte-derived MMPs may also cleave neuronal NrCAM (Fig. 6C).

### Validation of VCAM-1 and NrCAM cleavage in human samples

**Fig. 7.**
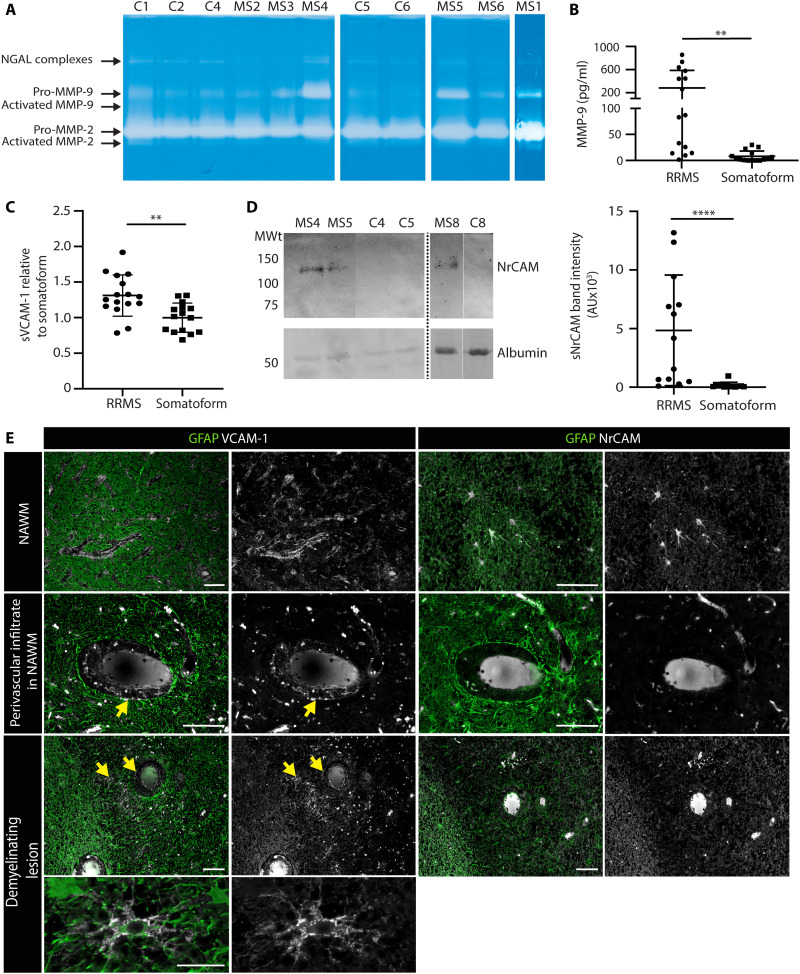
Identification of VCAM-1 and NrCAM in multiple sclerosis CSF samples and brain sections. (**A**) Representative gelatin gel zymography of CSF samples from patients with multiple sclerosis (MS1 to MS6) and age- and sex-matched somatoform controls (details in table S1). NGAL is neutrophil gelatinase–associated lipocalin. (**B**) ELISA for total MMP-9 in relapsing-remitting multiple sclerosis (RRMS) (*n* = 15) and somatoform (*n* = 15) CSF samples; statistical analyses were Mann-Whitney, **P < 0.005. The same CSF samples were tested in (**C**) ELISA for soluble VCAM-1 (sVCAM-1); data are expressed as change relative to somatoform controls. AU is arbitrary units. (**D**) Western blot for soluble NrCAM (sNrCAM); bar graph shows quantification of band intensities. Statistical analyses were Student’s *t* test (C) and Mann-Whitney (D); **P < 0.005 and ****P < 0.0001. (**E**) Double immunofluorescence staining for GFAP and VCAM-1 or NrCAM in normal-appearing white matter (NAWM), perivascular infiltrates in NAWM, and demyelinating lesions; scale bars, 100 μm. Arrows mark VCAM-1 expressed at the perivascular border and in the CNS parenchyma; bottom panels showing an astrocyte-expressing VCAM-1 in the demyelinating lesion at a higher magnification; scale bar, 25 μm.

To define whether cleavage of VCAM-1 and NrCAM also play a role in human tissues, we analyzed the CSF of different patients with multiple sclerosis and age- and sex-matched, non-multiple sclerosis somatoform controls (table S1) for gelatinase activity (Fig. 7, A and B) and the presence of soluble VCAM-1 or NrCAM. This revealed that elevated levels of MMP-9 were consistently associated with higher amounts of soluble VCAM-1 and soluble NrCAM detected (Fig. 7, C and D). Immunofluorescence staining of human biopsy material from patients with multiple sclerosis revealed VCAM-1 at cuff borders and on astrocytes in demyelinating lesions (Fig. 7E). Stainings for NrCAM showed similar patterns but were less intensive due to antibody quality; nevertheless, clear astrocyte NrCAM staining of some astrocytes was detected in multiple sclerosis samples.

## DISCUSSION

Using a tailored secretome MS approach, we identified previously unknown MMP-9 and MMP-2 substrates that are expressed by astroglial cells both in vitro and in vivo. The quantification of extracellular protein domains provided higher sensitivity for detection of shed proteins compared to standard analyses based on full-length proteins, validating our approach for the identification of protease cleavage substrates. While our approach detects extracellularly released protein regions independent of biochemical enrichments and is, therefore, particularly sensitive, elegant orthogonal strategies may further aid in the identification of explicit cleavage sites on protease substrates (*14*, *37*). As identifying gelatinase-derived protein fragments in brain extracts and relating them to the astroglial border is currently not possible, our secretome approach presents a powerful solution for ex vivo investigation of focal cleavage events.

Statistical analyses of proteins differentially shed by WT, DKO, and *Mmp2^−/−^* and *Mmp9^−/−^* cells revealed 16 common substrates for MMP-2 and MMP-9, which included largely cell-cell and cell-matrix molecules. As MMP-2 is constitutively expressed by astrocytes and MMP-2 and, in particular, MMP-9 are up-regulated in inflammation, such molecules are likely to be relevant for both homeostasis of the astroglia border and its breakdown in inflammation. Relatively few MMP-2 substrates were identified, several of which are likely to represent secreted vesicles or potentially exosomes, consistent with its constitutive expression and the role of astrocytes in communication between neurons and the endothelial barrier, which has been proposed to occur to some extent via exosomes and extracellular vesicles (*38*). By contrast, many potential MMP-9 substrates were identified, including cadherin-2, cadherin-4, cadherin-11, ADAM10, NrCAM, and VCAM-1, and some previously known substrates such as β-dystroglycan (*6*) and NOTCH (*9*) were confirmed, serving as internal controls and validation of our conceptual design. VCAM-1 and NrCAM were identified as gelatinase substrates at the astroglial border that were up-regulated during neuroinflammation, when barrier function is compromised, and were also detected in the CSF of patients with multiple sclerosis, suggesting relevance to changes in human BBB functional integrity.

Studies on the BBB have focused on the endothelial barrier and factors controlling its tightness, of which astrocytes and/or secreted factors thereof are known to play a central role (*39*). However, apart from influencing the endothelial barrier, the astroglial layer constitutes an independent barrier consisting of a layer of astrocytes and its associated BM. It is also known to communicate with the surrounding neurons, conveying their metabolic needs to the vasculature [reviewed in (*40*)]. The MS data provided here identified two main classes of molecules, affected by MMP-2/MMP-9 activity, that are likely to directly affect structural aspects of this astroglial border—cell-cell and cell-matrix adhesion molecules—but also suggest that gelatinase activity can affect communication between astrocytes and neurons.

An unexpectedly large number of cadherins [cadherin-2, cadherin-4, cadherin-11, cadherin19/cadherin-25, and calsyntenin-1 (CLSTN1)] or cadherin-associated molecules (PTPRK) were identified as potential MMP-9 substrates, most of which were validated at least at the gene level, which implies a more important role for adherens junctions at the astroglial border than considered to date. While adherens junction molecules have been reported to occur on astrocytes, particularly cadherin-2 (N-cadherin) (*41*), focus has been on gap junctions in astrocyte cell-cell communication (*42*), and in EAE, tight junctions have been reported to form between reactive astrocytes to stop infiltration of damaging serum factors into the brain parenchyma (*43*). There are some reports of MMPs cleaving tight junction molecules (*44*) and cadherins, including cadherin-2 (*45*); however, most are in vitro studies and do not correlate cleavage with an in vivo event or site of MMP action, and none of the studies used *Mmp2^-/-^* or *Mmp9^-/-^* mice. Together with our secretome data and in vivo location studies, the current data suggest that cell-cell junctions between astrocytes are dynamic structures that are focally tuned by gelatinase activity. Such focal remodeling of astrocyte-astrocyte interactions is likely to occur in inflammation where focal gelatinase activity also occurs (*6*, *9*) but may also be relevant to homeostasis and associated with constitutive astrocytic expression of MMP-2. Recent data have shown that microglia can extend processes between astrocytes to engage with the endothelium to modulate vascular function in homeostatic conditions (*46*, *47*). Given that both astrocytes and glia are sources of the gelatinases, penetration of the astroglia barrier may be facilitated by focal MMP activity and spatially limited disruption of junctions, which may be sufficient for penetration of glial processes without overt changes in barrier properties. Why astrocytes express so many different cadherins is an intriguing question and may be related to communication with multiple cell types including glia and perivascular cells. What controls focal gelatinase activity in homeostatic conditions is also not clear; both of these questions represent interesting directions for future investigations.

The identification of β-dystroglycan and its ligand, agrin, as well as several other ECM molecules (collagen VI and glypicans) as gelatinase substrates indicates that, in addition to cell junctions between astrocytes, astrocyte binding to the parenchymal BM also contributes to the barrier properties of the astroglial border, substantiating our earlier studies (*6*). Agrin has been reported to be cleaved by MMP-9 but not MMP-2 (*48*), as confirmed here. We have also previously investigated agrin’s expression in the parenchymal BM at sites of gelatinase activity and in whole brain extracts (*6*) but did not find evidence of its cleavage. The reason for this was probably the large amounts of protein in the whole-brain extracts and the focal mode of MMP action, resulting in very small amounts of cleaved agrin fragments in restricted areas. Identification here of agrin as a gelatinase substrate using our secretome, which was substantiated by our in vitro cleavage assay, further supports the sensitivity and strength of the secretome approach for a comprehensive analysis of protease cleavage substrates. As heparan sulfate proteoglycans, including agrin, act to link the laminin and collagen IV networks required for BM assembly and stability (*49*), the cleavage of agrin and of its receptor dystroglycan on astrocyte endfeet, is likely to compromise intermoleclular interactions within the parenchymal BM, making it more easy to penetrate, e.g., not only by infiltrating leukocytes in neuroinflammation (*6*) but also by probing microglia as discussed above (*46*, *47*). A similar role to dystroglycan may be played by the cell surface heparan sulfate proteoglycans, glypican-1, and glypican-4 (*50*), which are expressed on astrocytes and microglia and were identified here as to date unknown potential MMP-9 substrates. In addition, complexes at the surface of astrocyte endfeet that contain several of the here identified gelatinase substrates, including dystroglycan, agrin, and NrCAM (*51*), have been recently described, the significance of which remains unclear but is consistent with focal cleavage events at the astroglial border.

NrCAM has not been previously associated with alterations in BBB function; however, it has been reported to be cleaved by MMP-9 in olfactory stem cells (*36*). Its enhanced expression by astrocytes around inflammatory cuffs in EAE and detection of cleaved NrCAM in the CSF of patients with multiple sclerosis substantiate a role for this molecule at the astroglial border. Astrocytic NrCAM was recently shown to undergo homophilic interactions with neurons, promoting inhibitory synapse formation between neurons (*23*). Loss of such interactions by ablation of astrocytic NrCAM expression in mice significantly decreased inhibitory synapse function but had little effect on excitatory synapses (*23*). We show here that this is also the case when NrCAM is cleaved by the gelatinases, suggesting that focal gelatinase activity controls NrCAM-mediated astrocyte interactions with neurons. Mutations in NrCAM that would affect such gelatinase cleavage sites have been shown to have profound effects on behavior (*52*), consistent with behavioral changes in multiple sclerosis. These data suggest that NrCAM cleavage products and potentially also peptides derived from other gelatinase substrates identified here may not only affect the astroglial border directly but also have indirect effects on surrounding neurons or microglia.

The homophilic nature of NrCAM interactions further raises the possibility that it mediates astrocyte-astrocyte interactions and, thereby, contributes directly to astroglial barrier function. Consistent with this possibility, NrCAM deletion on astrocytes enhances astrocytic territory (*23*). Given that NrCAM was identified as a substrate of MMP-2, which is constitutively expressed in the CNS, and of MMP-9 that is expressed mainly in inflammation, suggests that this molecule may play a role in both homeostasis and neuroinflammation.

In addition to NrCAM, VCAM-1 was identified as an MMP-9 substrate that showed differential expression at the astroglial border during inflammation. Astrocytes were previously reported to express VCAM-1 under inflammatory conditions (*30*), and its expression requires TNF receptor signaling, which also promotes MMP-2/MMP-9 secretion and activation (*9*). In the absence of TNF receptor expression on astrocytes, VCAM-1 is no longer detectable and EAE does not result (*30*), similar to the situation when the gelatinases are ablated. However, its role on astrocytes has not been studied, and an astrocyte-specific VCAM-1 KO mouse has not been generated.

Cleaved VCAM-1 has been detected in human serum and CSF, with higher levels in multiple sclerosis (*53*); however, until now, the protease responsible for shedding was unknown. VCAM-1 is a classical immune cell adhesion molecule, which is known to interact with α4β1 on encephaliltogenic T cells to mediate binding to vascular endothelium in EAE. Targeting these interactions are the basis of a multiple sclerosis therapy, using an antibody to α4β1 (natalizumab) to inhibit entry of pathogenic T cells in the brain (*54*). Our data demonstrate that astrocyte VCAM-1 is also recognized by α4β1 on encephalitogenic T cells and that these interactions suppress proliferation and differentiation of the T cells toward pathogenic phenotypes, suggesting a gate-keeping role for astrocyte VCAM-1 at early stages of inflammation. These effects are, however, ablated by gelatinase cleavage of VCAM-1 upon breakdown of the astroglial barrier and leukocyte entry into the CNS parenchyma. The fact that anti-integrin α4β1, anti-VCAM-1, and soluble VCAM-1 inhibit encephalitogenic T cell adhesion to VCAM-1 on astrocytes and the suppressive effects on T cell differentiation indicate that cleaved VCAM-1 can compete with the cell-bound molecule, as suggested also by others (*55*), potentially displacing T cells bound at the astroglial border once the gelatinase concentration is sufficiently high (at late stages of EAE). This is likely to affect CD4^+^ T cell differentiation status and subsequent penetration of the parenchymal border.

In conclusion, by combining a tailored secretome approach with our in vivo knowledge on the astroglial barrier, we provide here a unique database of molecules that represent previously unknown potential gelatinase substrates that are likely to contribute to the barrier function of the astroglial border, the most prominent of which we have verified in in vitro and in vivo analyses. This both validates our conceptual approach to identifying proteolytic processes that control astroglial barrier function and provides possibilities for future research toward understanding the molecular nature of the astroglia barrier and its contribution to the BBB.

## MATERIALS AND METHODS

### Mice

C57BL/6 mice were from Charles River Laboratories (Cologne, Germany). *Mmp2^−/−^* (N11) (*56*) and *Mmp9^−/−^* (N9) (*57*) mice were on a C57BL/6 background. *Mmp2^+/−^ Mmp9^+/−^* females a nd *Mmp2^−/−^ Mmp9^−/−^* males were bred to generate DKO and single KO mice [*MMP2^+/−^/Mmp9^−/−^* (MMP-9 KO) and *Mmp2^−/−^/Mmp9^+/−^* (MMP-2 KO)]. Animal breeding and experiments were conducted according to the German Animal Welfare guidelines, permit number Az81-02.04.2018.A162 released by “Landesamt für Natur, Umwelt und Verbraucherschutz NRW,” Dusseldorf, Germany.

### MMPs and MMP inhibitors

Recombinant MMPs were from R&D Systems; *p*-aminophenylmercuric acetate (APMA; Sigma-Aldrich) was used to activate MMPs; MOBSAM, an *N*-sulfonylaminoacid derivative, is a dose-dependent inhibitor of MMPs (*18*) that was used for the specific inhibition of MMP-9 and/or MMP-2 (*9*).

### Primary astrocyte isolation and culture

Primary astrocyte cultures were prepared from the brains of newborn WT, *Mmp2^−/−^*, *Mmp9^−/−^*, and DKO mice (postnatal day 0 (P0) to P2) (*6*). Astrocytes were maintained in Dulbecco’s modified Eagle's medium (DMEM)/F12 medium supplemented with 20% fetal calf serum (FCS). Flow cytometry was performed using antibodies to GLAST, GFAP, and CD11b (table S2) to determine culture purity and the extent of microglia contamination.

### Gelatin gel zymography

The gelatinases were prepurified from astrocyte-conditioned medium (serum-free/overnight incubation) or undiluted human CSF samples using gelatin-Sepharose-4B (*6*) and separated on 10% polyacrylamide gels containing gelatin (1 mg/ml) under nonreducing conditions. Gels were washed in tris-buffered saline (TBS) and 2.5% Triton X-100, followed by TBS, 5 mM CaCl_2_, and 0.02% NP-40, and incubated overnight in the same buffer. Gels were stained with Coomassie blue and destained in acetic acid:methanol:H_2_O (10:50:40). Areas of cleaved gelatin appeared as clear bands that correspond to the proactivated and activated MMP subunits; recombinant mouse MMP-2 and MMP-9 were used as molecular weight controls.

### In situ zymography

To visualize gelatinase activity, fluorochrome-quenched (DQ)–gelatin (EnzCheck, Thermo Fisher Scientific) was used. Unfixed sections were covered with prewarmed reaction buffer (EnzCheck, Thermo Fisher Scientific) containing protease inhibitor–EDTA cocktail (Roche), DQ-gelatin (10 μg/ml), and gelatin (60 μg/ml), with or without 1,10-phenantrolin (50 mM; Sigma-Aldrich) for 1 hour at 37°C in a humid chamber. Sections were then washed, fixed in −20°C methanol, and immunofluorescence stainings were performed.

### Active EAE induction

Female mice (12 to 15 weeks of age) were immunized with MOG peptide (MOG_35–55_) (*5*) and monitored daily for weight loss and clinical symptoms. Disease severity was scored as stage 1 (flaccid tail), stage 2 (hindlimb weakness), stage 3 (severe hindlimb weakness), stage 4 (hind quarter paralysis), and stage 5 (forelimb weakness). All experiments were carried out as defined by the animal permit Az 81-02.04.2018.A162 released by Landesamt für Natur, Umwelt und Verbraucherschutz NRW, Dusseldorf, Germany.

### Collection of CSF

Mice were euthanized, and the head was mounted in a stereotaxic apparatus (ASI Instruments Inc., Warren, MI, USA). Skin, subcutaneous tissue, and muscles were removed to expose the cisterna magna. The cisterna magna was gently punctured using the micromanipulators, and CSF was collected using a Hamilton syringe (*58*).

### Immunofluorescence staining

Mice were euthanized at the peak of disease severity; isolated brains were embedded in Tissue-Tek O.C.T. and immediately frozen in liquid nitrogen; −20°C methanol-fixed 6-μm cryosections were used for most stainings. In some cases, brains were fixed overnight in 1% paraformaldehyde (PFA) at 4°C, embedded in 3% agarose, and thick sections (80 μm) were prepared using a Zeiss Vibratome.

Immunofluorescence staining was performed as described previously (*6*, *7*). Primary antibodies (table S2) were applied for 1 hour at room temperature (RT) in a humidified chamber or, in the case of thick sections, overnight at 4°C; secondary antibodies included donkey anti-rabbit AF594 (Invitrogen), Alexa Fluor 647 donkey anti-rat IgG (Abcam), Alexa Fluor 647 donkey anti-rabbit IgG (Abcam), Alexa Fluor 488 donkey anti-rat IgG (Invitrogen), and Alexa Fluor 594 goat ant-hamster IgG (Molecular Probes) and were applied for 60 min at RT; DAPI (4′,6-diamidino-2-phenylindole) (1 μg/ml) was used to visualize nuclei. Sections were examined with a Zeiss AxioImager or LSM700 confocal laser scanning microscope.

Cultured astrocytes were seeded onto Nunc Lab Tek chamber slides and grown to confluency. Cells were washed and fixed in −20°C methanol or, in the case of NOTCH3, in −20°C acetone for 10 min and blocked with 1% bovine serum albumin in PBS. Immunofluorescence staining was then performed as described above for thin sections.

### RNAscope

Organs were isolated under ribonuclease (RNase)–free conditions and immediately frozen; 13-μm cryosections were prepared and stored in RNase-free conditions for up to 3 months at −80°C. Sections or cultured astrocytes were fixed in cold RNase-free 4% PFA in PBS; rinsed; dehydrated at RT in 50, 70, and 100% ethanol; and stored in 100% ethanol at −20°C for up to a week or used directly. Sections/astrocytes were rehydrated at RT in 70 and 50% ethanol and PBS; RNAscope was carried out according to the manufacturer’s instructions using probes C1 (ADAM10 and VCAM-1). Hybridizations were carried out in the HybEZ oven and were followed by immunofluorescence staining for GFAP, pan-laminin, or CD45 as described above.

### Flow cytometry

Cultured astrocytes (0.5 × 10^6^ to 1.0 × 10^6^ cells per staining) were stained with directly labeled primary antibodies (table S2) diluted in fluorescence-activated cell sorting (FACS) buffer (2% FCS in PBS); isotype controls and unstained cells were used as negative controls. Cells were analyzed using a FACS Celesta. For intracellular staining, cells were permeabilized following staining for cell surface molecules using an intracellular staining kit (eBioscience); directly labeled primary antibodies or isotype controls (table S2) diluted in permeabilization buffer were used, and cells were analyzed as above.

### Western blot

Cultured astrocytes (1 × 10^6^ cells per well) were seeded onto six-well plates, cultured overnight without added cytokine or with TNF-α (100 ng/ml), washed, and lysed in TBS and 1% NP-40 plus protease inhibitor cocktail (Roche). Eighteen to 25 μg of total protein in Laemmli’s buffer ± β-mercaptoethanol was used per lane and separated on a 15% SDS-PAGE gel; for human CSF samples, 5 μl of CSF plus 15 μl of Laemmli’s buffer per lane was loaded and separated on a 10% SDS-PAGE gel. Mouse cerebellum extracts were in TBS and 1% NP-40 plus protease inhibitor cocktail (Roche); 50 μg of total protein was loaded per sample and separated on a 15% SDS-PAGE gel. Separated proteins were transferred to a nitrocellulose membrane (0.2-μm pore size, Whatman) using a Mini-PROTEAN Tetra Cell system (Bio-Rad). For dot blots of mouse CSF, 10 μl per sample was used. Membranes were washed, nonspecific binding was blocked, and membranes were incubated overnight at 4°C with primary antibodies (table S2). Bound antibodies were visualized using horseradish peroxidase (HRP)–labeled secondary antibodies [HRP goat anti-rat IgG + IgM (Dianova, 112-035-068), HRP donkey anti-sheep IgG (Millipore, AB324P), and HRP goat anti-mouse IgG (Bio-Rad, 170-6516)] and detected using chemiluminescence. Membranes were analyzed using a FUSION SL Gel Chemiluminescence Documentation system (Peqlab) and ImageJ software. Tubulin was used as a loading control; for human CSF samples, staining with Ponceau S and/or Coomassie was used as a loading control.

### In vitro cleavage

To check the ability of recombinant mouse MMP-9 and MMP-2 (R&D Systems) or recombinant mouse ADAM10 (R&D Systems) to in vitro cleave targets identified in the secretome analyses, mouse recombinant VCAM-1 (His-Tag) (Biozol), N-cadherin Fc-chimera (R&D Systems), cadherin-4 (R&D Systems), cadherin-11 Fc-chimera (R&D Systems), mouse recombinant NrCAM (R&D Systems), and recombinant rat agrin (R&D Systems) were diluted in 50 mM tris-HCl (pH 7.4), 200 mM NaCl, 5 mM CaCl_2_, 1 mM APMA, and 0.05% Brij35 to a final concentration of 40 μg/ml, and MMP-9 or MMP-2 was added to a final concentration of 4 μg/ml (10:1 ratio) or 400 ng/ml (100:1 ratio). Controls included MMP-9 or target proteins alone in assay buffer. ADAM10 cleavage assays were performed in 25 mM tris-HCl (pH 7.4), 2.5 μM ZnCl_2_, and 0.05% Brij35. Samples were incubated for 16 hours at 37°C; reactions were stopped by the addition of Laemmli buffer (β-mercaptoethanol); samples were separated on 10% SDS-PAGE gels, and protein bands were detected by silver staining.

### Sample preparation for secretome and full proteome analyses

Primary astrocytes from WT, *Mmp2^−/−^*, *Mmp9^−/−^*, and DKO mice (P0 to P2) were seeded into 12-well plates (0.5 × 10^6^ astrocytes per well) and cultured overnight. Cells were washed, Freestyle medium (Gibco) was added, and cells were incubated overnight at 37°C and 5% CO_2_. Conditioned media were collected, microcentrifuged to remove cell debris, snap-frozen in liquid nitrogen, and stored at −80°C until processed. WT astrocytes were either untreated or incubated overnight with 50 μM MOBSAM (Shionogi & Co.), which inhibits both MMP-2 and MMP-9 activation in a concentration-dependent manner (*18*). In all cases, eight replicates per condition or genotype were used. At the time of condition media collection, the viability of the cells was analyzed by flow cytometry, and GLAST/GFAP/CD11b expression was used to determine proportions of astrocytes.

Samples were denatured by adding 2% final SDS and 50 mM tris (pH 8.5); proteins were then reduced with dithiothreitol and alkylated with iodoacetamide as described previously (*15*). Acetone was added to conditioned media (final 80%, v/v) to precipitate proteins, and precipitates were solubilized with 2 M urea/10 mM Hepes and digested with trypsin (Promega) and LysC (Wako) for 16 hours at RT. Proteolytic digest was stopped by the addition of 0.5% (v/v) trifluoroacetic acid (TFA). Peptides were desalted on reversed-phase C18 stage tips and eluted with 80% (v/v) acetonitrile in 0.5% (v/v) acetic acid. Eluates were collected in PCR tubes and dried using a SpeedVac centrifuge (Eppendorf, Concentrator plus) at 60°C. Peptides were suspended in buffer A* [2% acetonitrile (v/v) and 0.1% TFA (v/v)] and sonicated (Branson Ultrasonics, Ultrasonic Cleaner Model 2510).

Full proteomes were analyzed for the different genotypes. For each genotype, 1 × 10^6^ cells were harvested and incubated in PreOmics lysis buffer for reduction of disulfide bridges, cysteine alkylation, and protein denaturation at 95°C for 10 min. Samples were sonicated [Bioruptor Plus from Diagenode (15 cycles, each of 30s)], and protein concentrations were determined using a tryptophan assay. In total, 200 μg of protein from each genotype was digested by adding trypsin and LysC (at a 1:100 ratio of enzyme to sample protein) and incubating at 37°C for 16 hours. The acidified peptides (1% TFA, v/v) were desalted on polystyrene-divinylbenzene, reversed-phase sulfonate (SDB-RPS) StageTips and eluted with 80% acetonitrile (ACN)/1.25% NH_4_OH. Eluates were collected in PCR tubes and dried using a SpeedVac centrifuge (Eppendorf, Concentrator plus) at 60°C. Peptides were suspended in buffer A* [2% acetonitrile (v/v) and 0.1% TFA (v/v)] and sonicated.

### Ultrahigh-performance LC and MS

Samples were analyzed by applying LC-MS instrumentation, comprising an EASY-nLC 1200 ultrahigh-pressure system (Thermo Fisher Scientific) coupled to a Q-Exactive HFX Orbitrap instrument (Thermo Fisher Scientific) with a nanoelectrospray ion source (Thermo Fisher Scientific).

Peptides were loaded on a 50-cm reversed-phase column [75-μm inner diameter, packed in-house with ReproSil-Pur C18-AQ 1.9-μm resin (Dr. Maisch GmbH)]. The column temperature was maintained at 60°C using a homemade column oven. An EASY-nLC 1200 system (Thermo Fisher Scientific) was directly coupled online with the mass spectrometer (Q-Exactive HF-X, Thermo Fisher Scientific) via a nanoelectrospray source, and peptides were separated with a binary buffer system of buffer A [0.1% formic acid (FA)] and buffer B (80% acetonitrile plus 0.1% FA), at a flow rate of 300 nl/min. Peptides were eluted with a 45-min gradient of 5 to 60% buffer B [0.1% (v/v) FA and 80% (v/v) ACN]. After each gradient, the column was washed with 95% buffer B for 5 min. MS data were acquired with a Top15 data-dependent MS/MS scan method (topN method). Target values for the full-scan MS spectra were 3 × 10^6^ charges in the 300 to 1650 mass/charge ratio (*m*/*z*) range with a maximum injection time of 55 ms and a resolution of 120,000 at 200 *m*/*z*. Fragmentation of precursor ions was performed by higher-energy C-trap dissociation with a normalized collision energy of 27. MS/MS scans were performed at a resolution of 15,000 at 200 *m*/*z* with an ion target value of 5 × 10^4^ and a maximum injection time of 25 ms.

### LC-MS/MS data analysis

Protein identification and quantification from MS raw files were performed using the computational proteomics platform MaxQuant (version 1.6.2.1) (*19*). Murine MS/MS spectra were searched against the respective UniProt FASTA databases and a common contaminant database by the implemented Andromeda search engine (*59*). Trypsin/P with a minimum peptide length of seven amino acids and a maximum of two missed cleavages was used as digestion mode. Cysteine carbamidomethylation was set as fixed and methionine oxidation and N-terminal acetylation were set as variable modifications. False discovery rates were 1% at the peptide and protein level. Peptide identification was performed with an allowed initial precursor mass deviation up to 4.5 parts per million (ppm) and an allowed fragment mass deviation of 20 ppm. Protein identifications required one unique or razor peptide. For nonlinear retention time alignment of all samples, the “Match between runs” option of MaxQuant was used. The time windows for matching peptide identifications across different samples and to search for the best alignment function were set to 0.7 and 20 min. For LFQ via MaxLFQ, a minimum ratio count of 1 was used (*17*).

### MMP-2 and MMP-9 substrate identification

To identify MMP-9 and MMP-2 substrates, peptide intensities were logarithmized, peptides were filtered for 70% valid values in at least one group, and proteins with less quantification were excluded from further analysis. Because of no prior normalization of peptide intensities in MaxQuant, quantile normalization was performed in Perseus (option: with adjustment) to adjust for potential loading differences in the samples (*60*). Peptides were filtered by the UniProt keyword “membrane” (excluding purely cytoplasmic proteins, keyword: cytoplasm) to focus subsequent analysis on relevant MMP target proteins, and the missing values were imputed from normal distribution (width: 0.3, downshift: 1.8).

The following analyses were performed in the R environment, and tailored algorithms were developed. Peptides were annotated “extracellular” and “intracellular” via UniProt keywords, respectively, or “not specified” if neither was applicable. Proteins belonging to the extracellular matrix, for example, are not annotated with “intracellular” or “extracellular” domains, but might be targets of the extracellular active MMPs and thereby receive an annotation “not specified,” to not exclude them. For the following comparisons, all peptides localized in the respective protein domain—extracellular and not-specified domains were grouped together, intracellular was kept separately—were protein-wise grouped together and compared between the different genotypes/cell inhibition conditions. Separate two-tailed *t* tests were performed for extracellular/not specified domains and intracellular domains to statistically compare different genotypes and small-molecule treatments, respectively. Raw files were deposited in the Pride repository.

### In vitro CD4^+^ T cell adhesion to astrocytes

Astrocytes seeded onto eight-well Lab-Tek chamber slides were activated overnight with TNF-α (100 ng/ml). Primary encephalitogenic CD4^+^ T cells were isolated from spleens and lymph nodes of three EAE mice using the CD4^+^ magnetic beads (magnetic-activated cell sorting) selection kit (Miltenyi Biotec). CD4^+^ T cells were stained with 5(6)-carboxytetramethylrhodamine, succinimidyl ester (TAMRA) (10 μg/ml; Thermo Fisher Scientific) and 1 × 10^6^ cells were added per well in adhesion buffer (DMEM, 5% FCS, 2% l-glutamine, and 25 mM Hepes). CD4^+^ T cells were either untreated or preincubated for 15 min at RT with anti-mouse integrin α4 antibody (PS/2, 20 μg/ml; Pharmingen) or recombinant murine VCAM-1 (4 μg/ml; R&D Systems) before addition to the astrocytes. In some cases, astrocytes were incubated for 1 hour at RT with anti-mouse VCAM-1 antibody (M/K-2, 20 μg/ml; table S2) in adhesion buffer. CD4^+^ T cells were incubated with astrocytes for 30 min at RT on a shaker; three to four technical replicates per condition were used. Wells were washed, fixed with 1.25% glutaraldehyde, and analyzed for TAMRA^+^-adherent T cells using a Zeiss AxioImager; images were captured using a Hamamatsu ORCA-ER camera and analyzed using Volocity 5.2 software (ImproVision) and ImageJ.

### In vitro proliferation assay

Encephalitogenic CD4^+^ T cells were isolated from lymph nodes and spleens of WT mice on day 8 after EAE induction using CD4^+^ selection with magnetic beads (Miltenyi Biotec). T cells were stained with CellTrace violet dye (Thermo Fisher Scientific), and the initial cell trace staining intensity was measured using flow cytometry (FACS Celesta, Beckton Dickinson). CD4^+^ T cells (0.25 × 10^6^) were plated alone (unstimulated control) or together with 0.05 × 10^6^ CD11c^+^ dendritic cells and MOG_35–55_ peptide (20 μg/ml) for 3 days, either without substrate (control), with soluble recombinant mouse VCAM-1 (10 μg/ml; Biozol), or plated onto coated VCAM-1 (10 μg/ml). The extent and symmetry of cell division were monitored by cell trace dilution analysis using flow cytometry.

### In vitro differentiation assay

For differentiation experiments, the encephalitogenic T cells were isolated as for the proliferation assay. In addition, to induce differentiation, subset-specific cytokines and antibodies [interleukin-12 (IL-12) for T_H_1; IL-6, transforming growth factor–β, and anti–interferon-γ for T_H_17] were added to the cocultures of CD4^+^ T cells and CD11c^+^ dendritic cells. Culturing conditions included no substrate or added factors (control), with soluble VCAM-1 (10 μg/ml), and cells plated onto coated VCAM-1 (10 μg/ml) or anti-integrin α4 antibody (20 μg/ml; PS/2). After 4 days of culture, cells were restimulated using phorbol 12-myristate 13-acetate/ionomycin for 4 hours, fixed, permeabilized, and stained for intracellular subset-specific cytokines (BioLegend) and analyzed by flow cytometry.

### Polymerase chain reaction

RNA was isolated from cultured astrocytes using the RNeasy Mini Kit (Qiagen) according to the manufacturer’s instructions. Complementary DNA was transcribed from 2 μg of RNA using the Omniscript RT Kit (Qiagen). Primers (Metabion, Steinkirchen, Germany) used in PCR analyses are listed in table S3.

### Astrocyte/neuronal cocultures (synapse development)

Dissociated cultures of primary hippocampal neurons and astrocytes were prepared from newborn mice and plated onto Matrigel-covered glass coverslips in plating medium [MEM, 10% FCS, 2 mM l-glutamine, glucose (5 g/liter), NaHCO_3_ (0.2 mg/ml), transferrin (0.1 mg/ml), and insulin (0.025 mg/ml)]. Starting 2 days after plating, the medium was successively exchanged to growth medium (Neurobasal A medium (NBA), 2% B27, 2 mM GlutaMAx, and 2 μM cytosine arabinoside). Conditioned media were collected on day 15 of coculture for analysis in gelatin gel zymography.

Immunostaining of primary hippocampal neurons and astrocytes was performed on day 15 of culture. Neurons were fixed (4% PFA and 4% sucrose in PBS) for 20 min at RT, followed by incubation for 1 hour at 37°C in blocking buffer (PBS, 0.1 M glycine, 0.2% saponin, and 10% goat serum). Subsequently, cells were incubated for 1 hour at RT with rabbit anti-vGAT, mouse anti-vGlut1, and guinea pig anti-GFAP (table S2) in antibody buffer (PBS, 0.1 M glycine, 0.2% saponin, and 5% goat serum). After washing (PBS, 0.1 M glycine, 0.05% saponin, and 5% goat serum), cells were incubated with the secondary antibodies (goat anti-mouse Alexa Fluor 488, goat anti-rabbit Alexa Fluor 568, and goat anti–guinea pig Alexa Fluor 647; Thermo Fisher Scientific) in antibody buffer for 1 hour at RT and mounted in Mowiol.

Samples were imaged with a laser scanning confocal microscope (TCS SP8 microscope, Leica Microsystems) using a 40× oil immersion objective (numerical aperture of 1.0). The zoom factor was set to result in a pixel size of 200 nm. Quantitative analysis was performed with self-written transformation (*61*) with the levels *k* = 3 and *l*_d_ = 1, resulting in a segmented mask image. The number of spots on the mask images, each representing putative presynaptic boutons, were quantified for the individual detection channels. Only spots with areas between 6 and 50 pixels were accepted.

### Staining for VCAM-1 and NrCAM in human multiple sclerosis tissue

We retrospectively analyzed paraffin-embedded brain tissue samples from two patients with multiple sclerosis, which were histologically characterized by inflammatory demyelination, consistent with the histological diagnosis of multiple sclerosis. Lesions were classified as active lesions (*62*). The study was approved by the Ethics Committee of the University of Münster (AZ2012-407-f-S and 2016-165-f-S) (table S1). Sections were deparaffinized using Xylol and then 100, 96, and 70% ethanol. To demask epitopes, tissues were pretreated for 40 min in a steamer in citric buffer (pH 6) for NrCAM staining or tris-EDTA buffer (pH 9) for VCAM-1 staining, followed by 15 min at RT. After a 20-min incubation in 10% FCS in PBS, primary antibodies (table S2) were applied overnight at 4°C in a humidified chamber; secondary antibodies were goat anti-rabbit Cy3 (Dianova) and Alexa Fluor 647 goat anti-mouse IgG (Invitrogen), which were applied for 60 min at RT; directly labeled GFAP (table S2) was stained afterward for 60 min at RT; DAPI (1 μg/ml) was used to visualize nuclei. Sections were examined with a Zeiss AxioImager microscope.

### ELISA for soluble VCAM and MMP-9 in human CSF

The human sVCAM-1/CD106 Quantikine enzyme-linked immunosorbent assay (ELISA) kit (R&D Systems) was used according to the manufacturer’s instructions to determine concentrations of soluble VCAM-1 in the CSF of patients with multiple sclerosis versus non-multiple sclerosis (somatoform) controls. To determine the concentration of total MMP-9 in the CSF of patients with multiple sclerosis versus non-multiple sclerosis (somatoform) controls, the human MMP-9 DuoSet ELISA Kit (R&D Systems) was used according to the manufacturer’s instructions. The study was approved by the Ethics Committee of the University of Münster (2019-712-f-S, 2016-053-f-S, and 2010-262-f-S).

### Statistical analysis

Statistical analyses were performed using the GraphPad Prism 7.0 software (GraphPad Inc., La Jolla, CA, USA). Data were tested for normal distribution, and either nonparametric Mann-Whitney test, Student’s *t* test, or two-way analysis of variance (ANOVA) was used. Statistical significance was assumed for *P* values < 0.05.

## Notes

View/request a protocol for this paper from *Bio-protocol*.
