# Supplementary material for: Secretomics reveals gelatinase substrates at the blood-brain barrier that are implicated in astroglial barrier function
Source: Sci Adv. 2023 Jul 19;9(29):eadg0686. doi: 10.1126/sciadv.adg0686 (PMC10355830; doi:10.1126/sciadv.adg0686)
Supplement: Supplementary file 1 — Figs. S1 to S13 Tables S1 to S3 [file sciadv.adg0686_sm.pdf]

Science Advances
AAAS

Supplementary Materials for

Secretomics reveals gelatinase substrates at the blood-brain barrier that are implicated in astroglial barrier function

Miriam Burmeister *et al.*

Corresponding author: Lydia Sorokin, sorokin@uni-muenster.de; Felix Meissner, felix.meissner@uni-bonn.de

Sci. Adv. **9**, eadg0686 (2023)
DOI: 10.1126/sciadv.adg0686

This PDF file includes:

Figs. S1 to S13
Tables S1 to S3

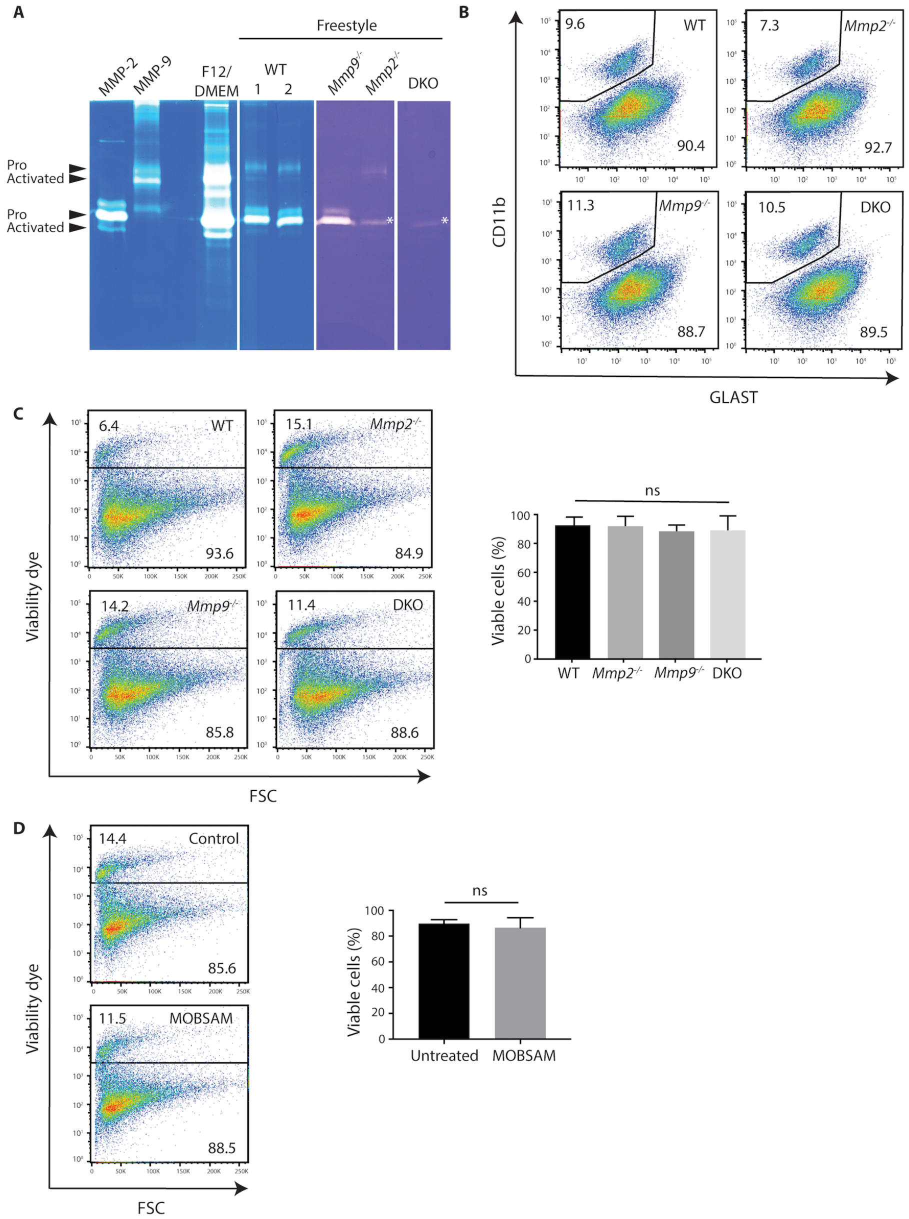

Fig. S1 Characterisation of astroglial cultures employed for mass spectrometry secretome analyses. (A) Gelatin gel zymography of conditioned media from 1x10^6 astroglial cells cultured overnight in serum-containing F12/DMEM medium or in serum-free Freestyle medium; WT 1 and 2 refer to two different samples; asterisk mark band arising from low levels of serum contamination (0.05%). (B) Flow cytometry of WT, *Mmp9*$^{-/-}$, *Mmp2*$^{-/-}$ and DKO astroglial cells cultured in Freestyle medium to determine proportions of astrocytes (GLAST$^+$ CD11b^{neg}) versus glial cells (GLAST^{neg} CD11b$^+$) and (C) corresponding viability of the cells, as defined by exclusion of viability dye; bar graph shows quantification of viable cells in the four conditions employed; data are means ± SD from experiments performed with 4 replicates. (D) Flow cytometry assessment of cell viability of astrocytes cultured in the presence of the MMP inhibitor MOBSAM (50 μM); data was analysed as in (C) except that 8 replicates were employed. Statistical analyses were one-way ANOVA test (C) or unpaired T-test (D).

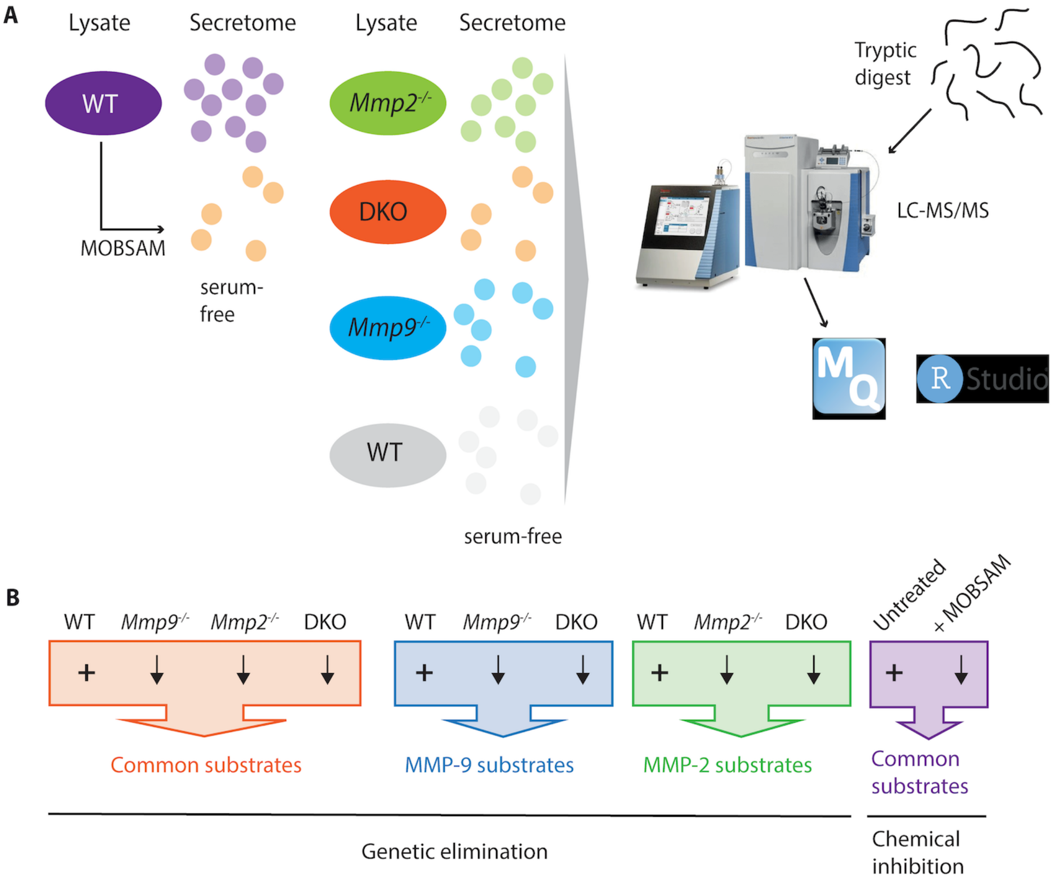

Fig. S2 Experimental approach using different MMP knockout samples and chemical MMP inhibition with MOBSAM to determine MMP substrates. (A) Workflow consisting of collection and processing of conditioned media (secretome) and cell lysates (lysates), sample preparation, LC-MS/MS analysis and data processing. (B) Strategy to further identify MMP-2 and MMP-9 substrates.

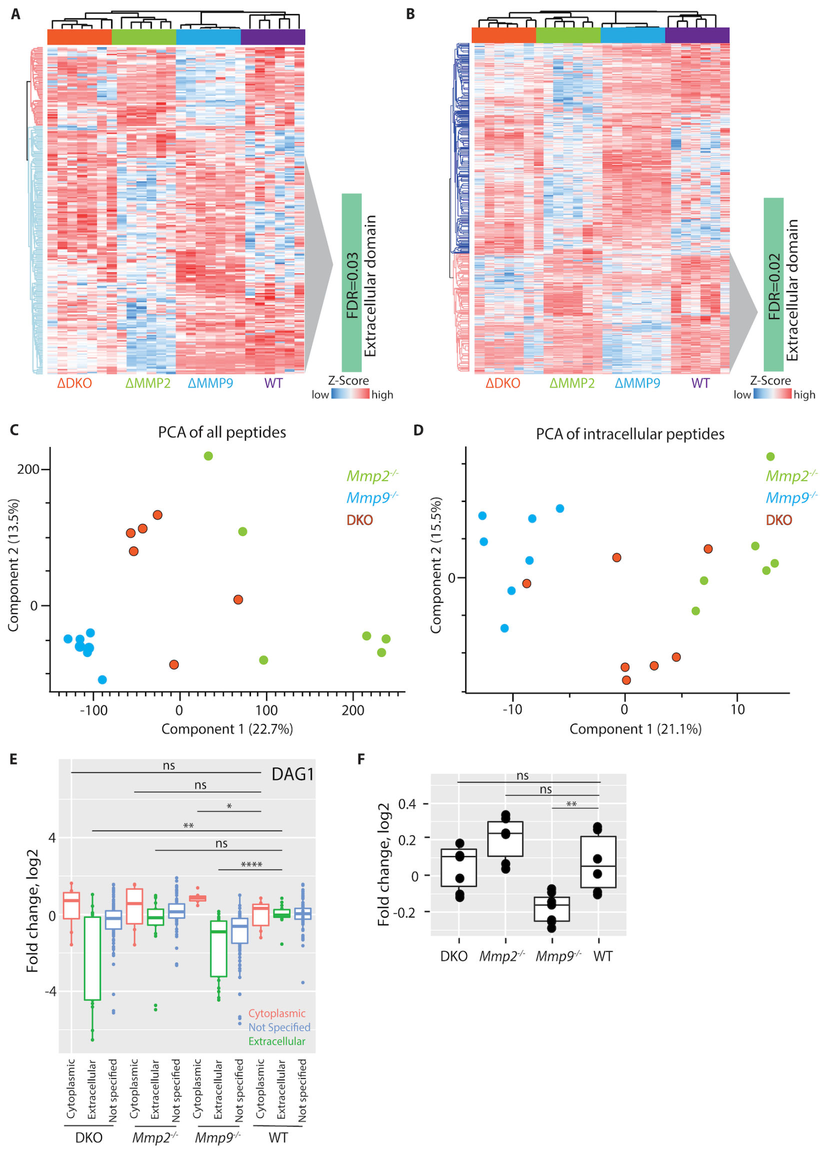

Fig. S3 Differentially shed proteins upon genetic MMP ablation. (A) Hierarchical clustering (Pearson correlation) of significantly regulated peptides of potential MMP-2 substrates, showing differential enrichment of extra-cellular domains in one of the two major clusters. (B) Hierarchical clustering (Pearson correlation) of significantly regulated peptides of potential DKO substrates, showing differential enrichment of extra-cellular domains in one of the two major clusters. (C) Principal component analysis (PCA) of all proteins (40 746 peptides corresponding to 4943 proteins) from *Mmp2*$^{-/-}$, *Mmp9*$^{-/-}$ and DKO conditioned media normalised to WT. Component 1 accounts for 22.7% of the data variation. (D) Principal component analysis (PCA) of the intracellular protein domains of proteins with membrane annotation (320 peptides corresponding to 110 proteins) from *Mmp2*$^{-/-}$, *Mmp9*$^{-/-}$ and DKO conditioned media normalised to WT. Component 1 accounts for 21.1% of the data variation. (E) Regulation of the extracellular (green), not specified (blue) and intracellular (red) domains of DAG1 for the different genotypes quantified with the localization specific quantification. (F) Regulation of DAG1 quantified with LFQ. Each boxplot represents six independent measurements; Student's T-test; *p<0.05, **p<0.01, ****p<0.0001.

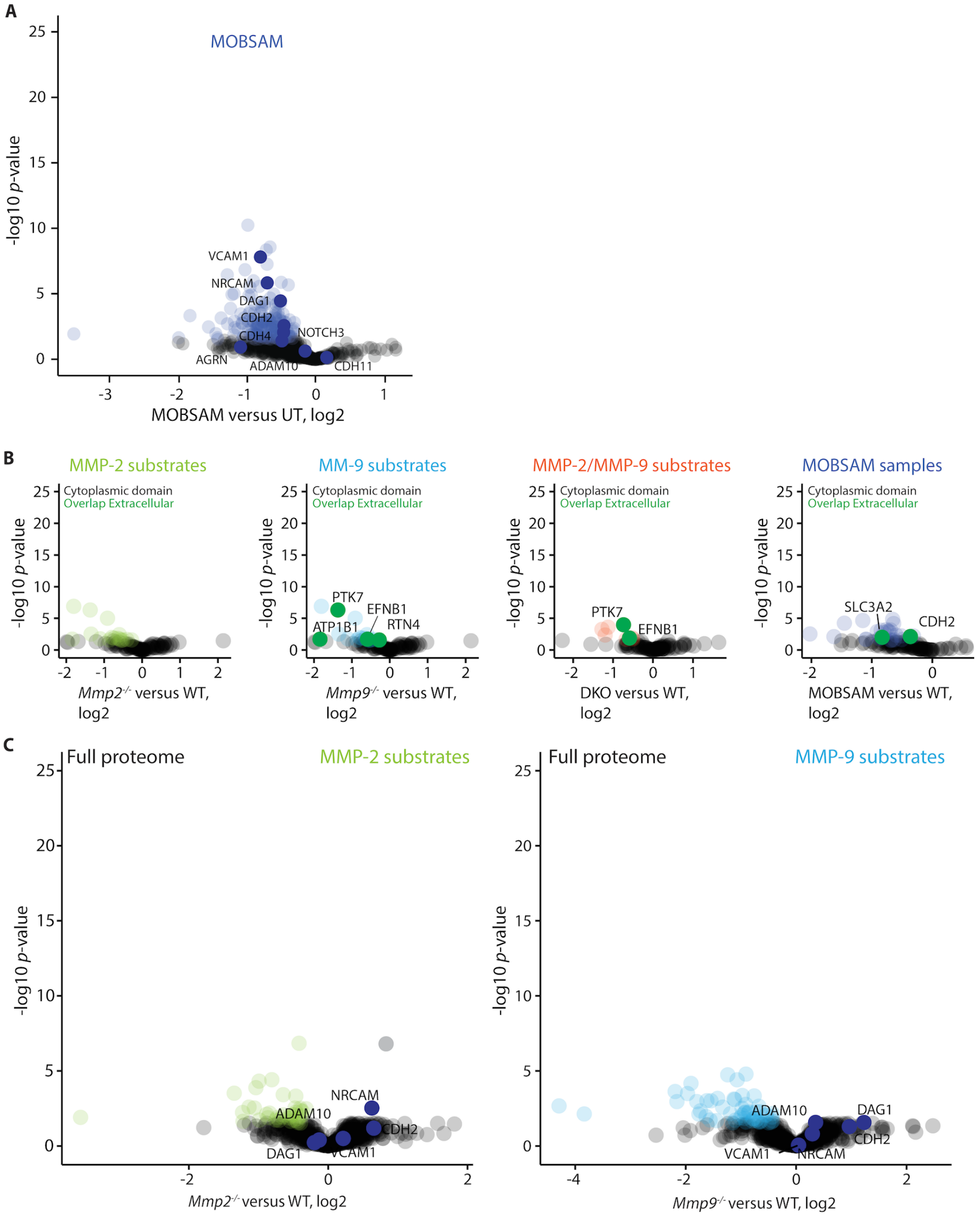

Fig. S4 Secretome analysis of astrocyte conditioned media (A) Volcano plot showing differentially changing extracellular and not specified protein domains detected in the conditioned media. Pairwise comparison to define MOBSAM specific substrates (dark blue, p<0.03). p-values (-log10) are plotted against the difference (log2) between the chemical inhibition and WT (Student's T-test). (B) Volcano plots showing differentially changing intracellular domains detected in the different MMP conditioned media. Pairwise comparisons to define MMP-2 (light green, p<0.03), MMP-9 (light blue, p<0.03), DKO (orange, p<0.03) and MOBSAM (dark blue, p<0.03) regulated proteins. Overlaps of potential MMP substrates are plotted in dark green. (B) Volcano plots showing differentially changing extracellular domains of membrane annotated proteins detected in the cell lysates. Pairwise comparisons to define MMP-2 (light green, p<0.03), MMP-9 (blue, p<0.03) genotype specific substrates. Overlaps of potential MMP substrates are plotted in dark green.

A

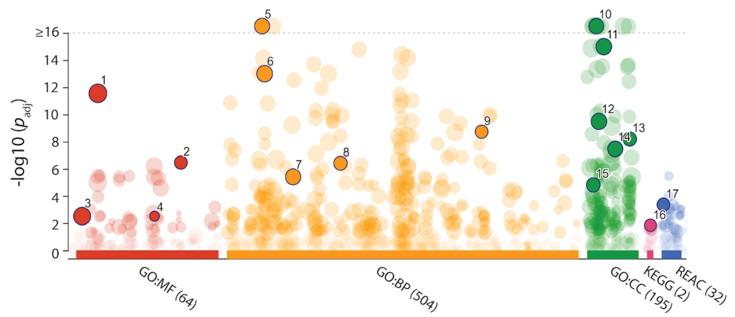

ID	Source	Term Name	p_{adj} (query_1)
1	GO:MF	Protein binding	2.814×10^{-12}
2	GO:MF	Cell adhesion molecule binding	3.472×10^{-7}
3	GO:MF	Catalytic activity	3.085×10^{-3}
4	GO:MF	Laminin binding	3.078×10^{-3}
5	GO:BP	Cell adhesion	1.200×10^{-17}
6	GO:BP	Nervous system development	1.004×10^{-13}
7	GO:BP	Vesicle-mediated transport	3.914×10^{-6}
8	GO:BP	Cell junction assembly	3.928×10^{-7}
9	GO:BP	Cell-cell adhesion via plasma-membrane adhesion	1.881×10^{-9}
10	GO:CC	Cell surface	4.839×10^{-18}
11	GO:CC	Vesicle	1.016×10^{-15}
12	GO:CC	Cell junction	3.257×10^{-10}
13	GO:CC	Glutamatergic synapse	6.475×10^{-9}
14	GO:CC	Synapse	3.448×10^{-8}
15	GO:CC	Cell-cell junction	1.521×10^{-5}
16	KEGG	Cell adhesion molecules	1.426×10^{-2}
17	REAC	Axon guidance	4.340×10^{-4}

B

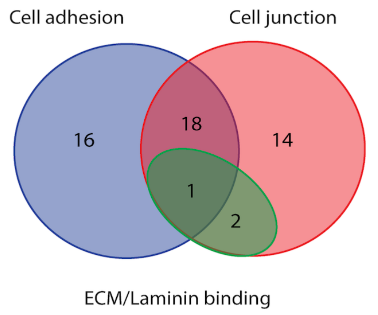

Cell adhesion + Cell junction + ECM/ laminin binding	DAG1
Cell adhesion + Cell junction	CDH2, PTPRS, NRCAM, PTPRK, CLSTN1, IQGAP1, DCHS1, PTK7, GPC4, ADAM10, EXT1, EFNB1, MXRA8, MYADM, EGFR, FGFRL1, NRP1, B4GALT1
Cell junction + ECM/laminin binding	AGRN, GPC1
Cell adhesion	ATP5B, CDH4, NCAM1, CHL1, VCAM1, ADAM12, ICOSLG, CD200, GLYCAM1, COL6A, AXL, LRRC4B, IL4R, RTN4, MCAM, MMP14
Cell junction	VDAC2, VDAC1, DLGAP4, CAP2, ITSN1, AP2B1, LRRC4B, GNAQ, AP2A2, RTN4, ATP6AP2, CXADR, CDH11, FLRT3

Fig. S5 Functional enrichment analysis of potential MMP-9 targets using g:Profiler. (A) Manhattan plot illustrating enrichment analysis results. Highly significant functional terms and functional terms of interest are highlighted. Adjusted enrichment *p*-values (p_{adj}) are plotted on the Y-axis against the data sources on the X-axis. GO=Gen Ontology, MF=Molecular Function, BP=Biological Property, CC=Cellular Component, KEGG= Kyoto Encyclopedia of Genes and Genomes, REAC= Reactome. (B) Venn diagram of potential MMP-9 substrates associated with cell junctions and/or cell adhesion and/or ECM/laminin binding. Potential substrates are listed on the right, sorted based on their intersection.

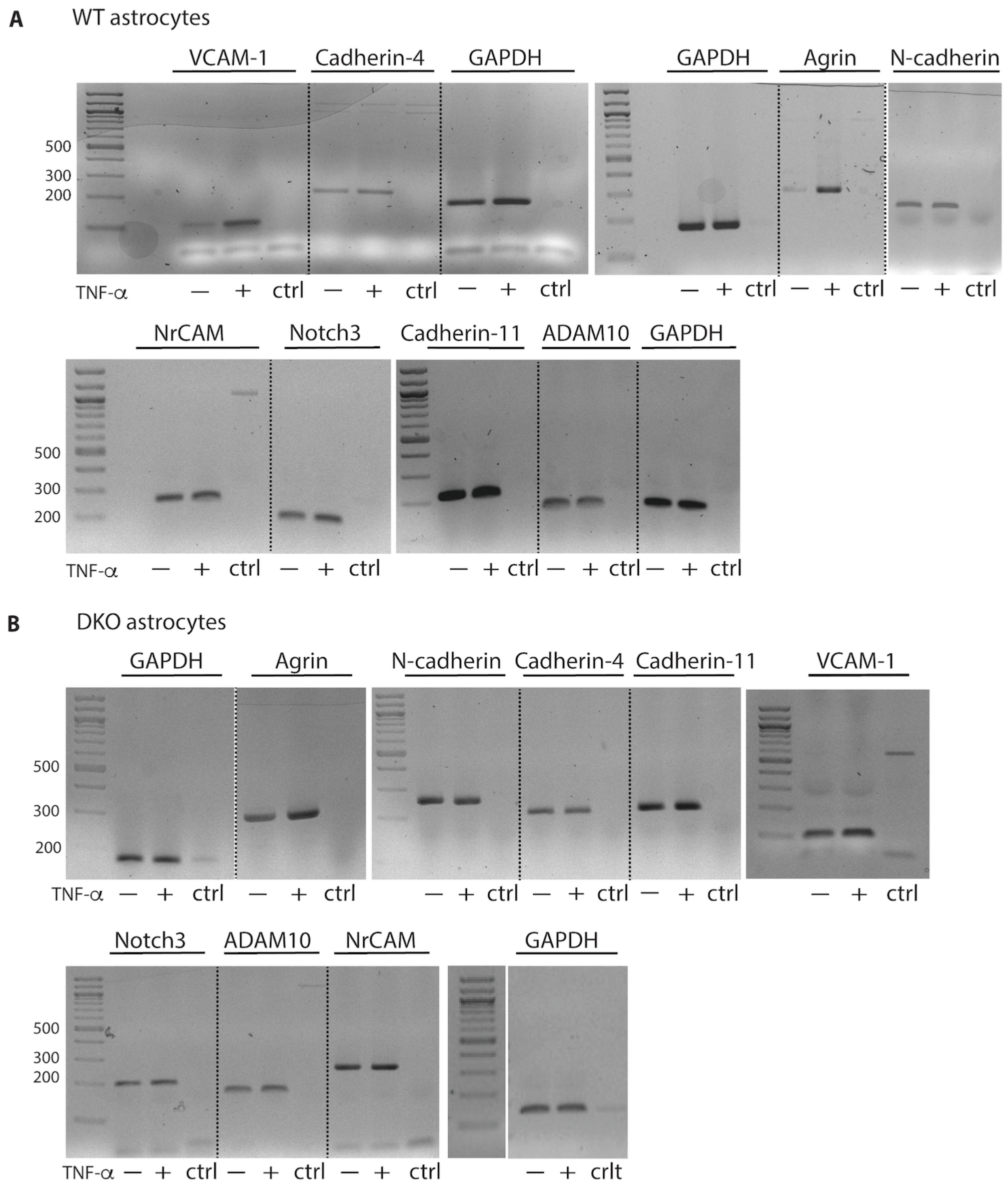

Fig. S6 PCR for detection of gelatinase substrates in (A) WT and (B) DKO cultured astroglial cells under non-stimulated (-) and TNF-α stimulated (+) conditions; ctrl is water control.

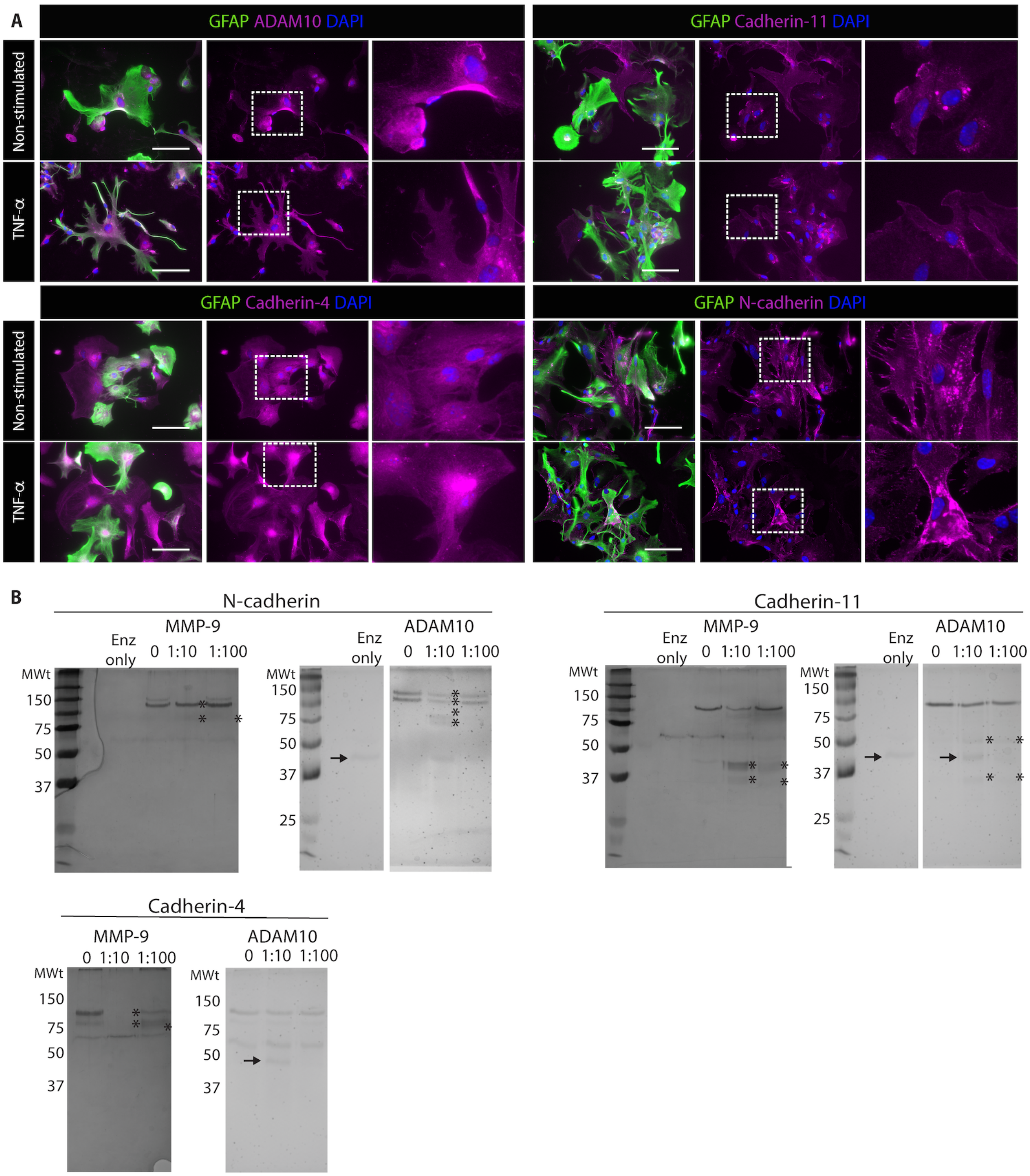

Fig. S7 Expression and cleavage of selected gelatinase substrates in WT astroglial cultures. (A) Representative double immunofluorescence stainings for MMP substrates and GFAP in non-stimulated and TNF-α stimulated astroglial cultures; boxed areas are shown to the right at higher magnifications; scale bars are 100 μm. (B) Silver stained gels showing cleavage products of gelatinase substrates after overnight incubation with 1:10 or 1:100 ratios of MMP-9:substrate or ADAM10:substrate; arrows mark the positions of ADAM10 in samples; asterisks mark specific cleavage products. Data shown are representative of 2-3 experiments.

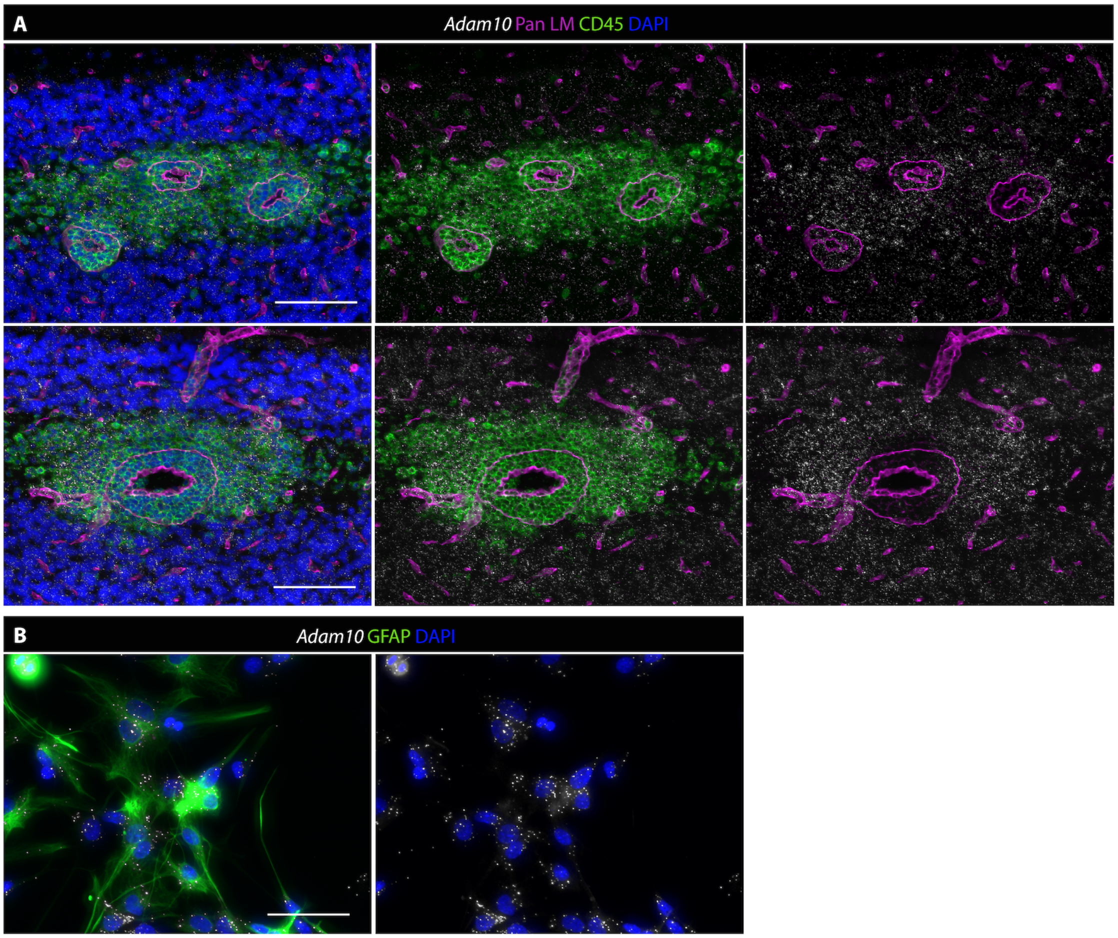

Fig. S8 RNAscope for *Adam10* mRNA *in vivo* and *in vitro*. (A) RNAscope using *Adam10* specific probe was performed on EAE brain sections and costained for CD45 and pan-laminin (Pan LM) to visualize perivascular cuffs and the extent of leukocyte infiltration; two different areas with different sized cuffs are shown in upper and lower panels; scale bars are 100 μm. (B) RNAscope for *Adam10* was performed on the astroglial cultures, and costained for GFAP to mark astrocytes, scale bar is 50 μm; DAPI staining was employed to mark all nuclei.

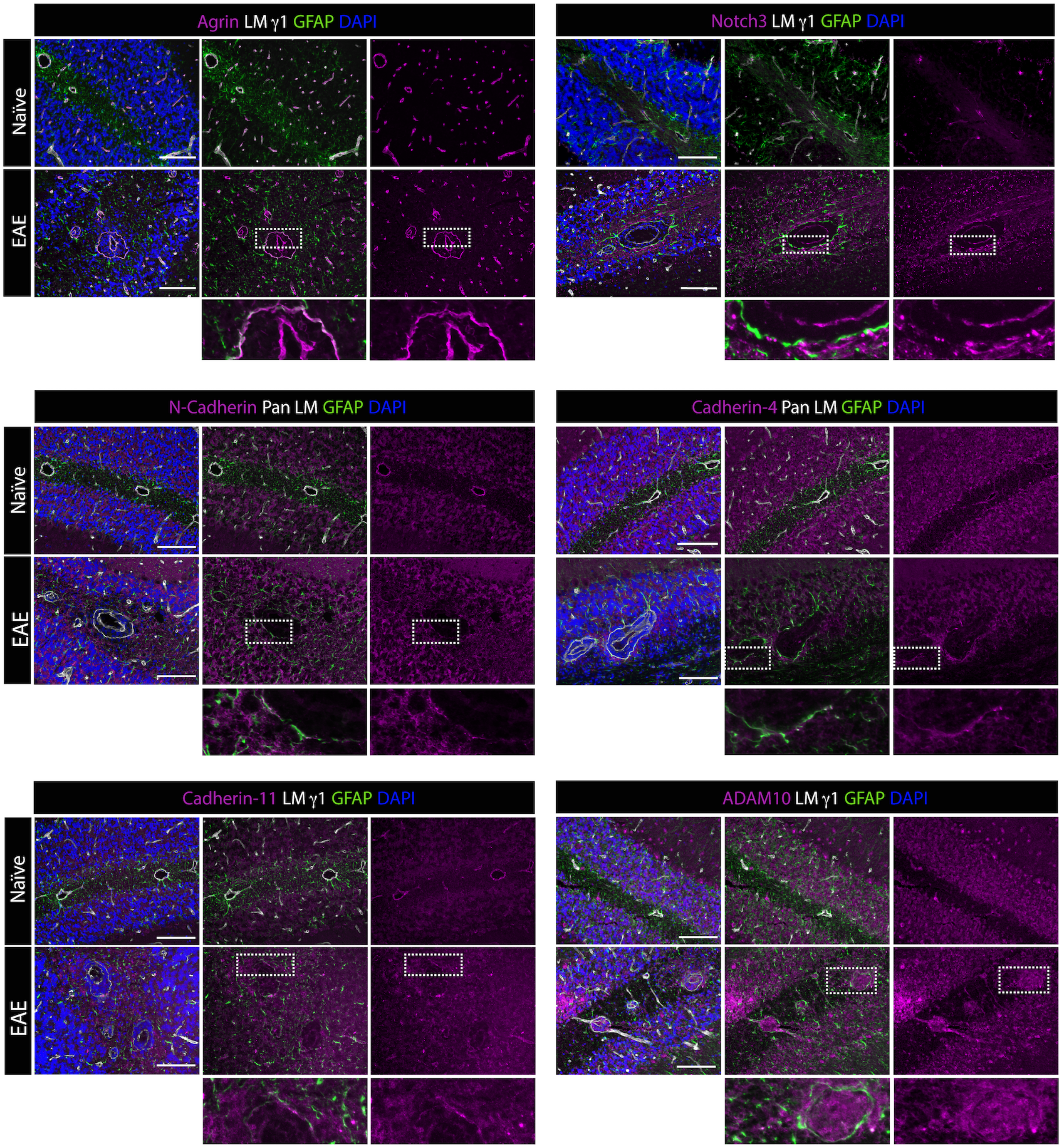

Fig. S9 *In vivo* confirmation of the MMP substrates in non-inflamed (naïve) and EAE brains. WT brain sections were triple immunofluorescently stained for Agrin, NOTCH3, N-cadherin, cadherin-4, cadherin-11 or ADAM10, plus GFAP to mark astrocytes, and pan-laminin (Pan LM) or laminin γ1 chain to mark basement membranes and perivascular inflammatory cuffs; DAPI marks all nuclei; areas outlined by dotted lines are shown at higher magnifications in lower panels. Scale bars are 100 μm.

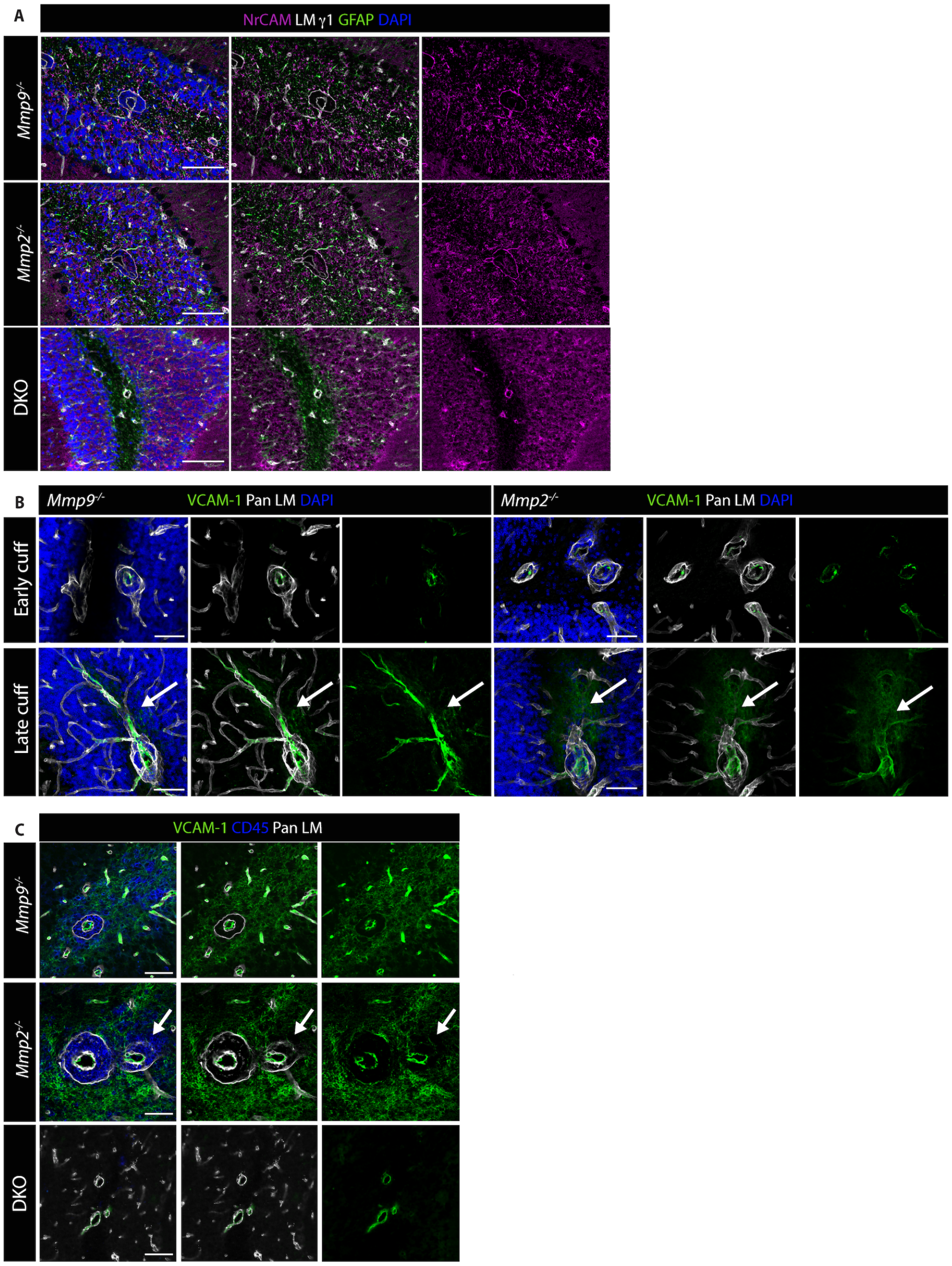

Fig. S10 ***In vivo*** **expression of MMP substrates, NrCAM and VCAM-1, in** ***Mmp2$^{-/-}$*****,** ***Mmp9$^{-/-}$*** **and DKO EAE brains.** *Mmp2$^{-/-}$*, *Mmp9$^{-/-}$* and DKO brain sections were immunofluorescently stained for (A) GFAP to mark astrocytes, anti-laminin γ1 chain antibody to mark basement membranes (BM) and perivascular cuffs, and NrCAM; DAPI marks all nuclei. DKO do not develop EAE symptoms and lack inflammatory cuffs. Scale bars are 100 μm. (B) Immunofluorescence staining for VCAM-1 and pan-laminin (Pan LM) in early and late stage EAE brains; arrow marks VCAM-1 in CNS parenchyma at late stage EAE; DAPI marks nuclei; scale bars are 50 μm. (C) Triple immunofluorescence staining for CD45, Pan LM and VCAM-1 shows upregulation of VCAM-1 around inflammatory cuffs and its loss at this site where CD45 infiltration occurs (arrows) in *Mmp2$^{-/-}$*, consistent with its cleavage mainly by MMP-9; DKO do not develop inflammatory cuffs. Scale bars are 50 μm.

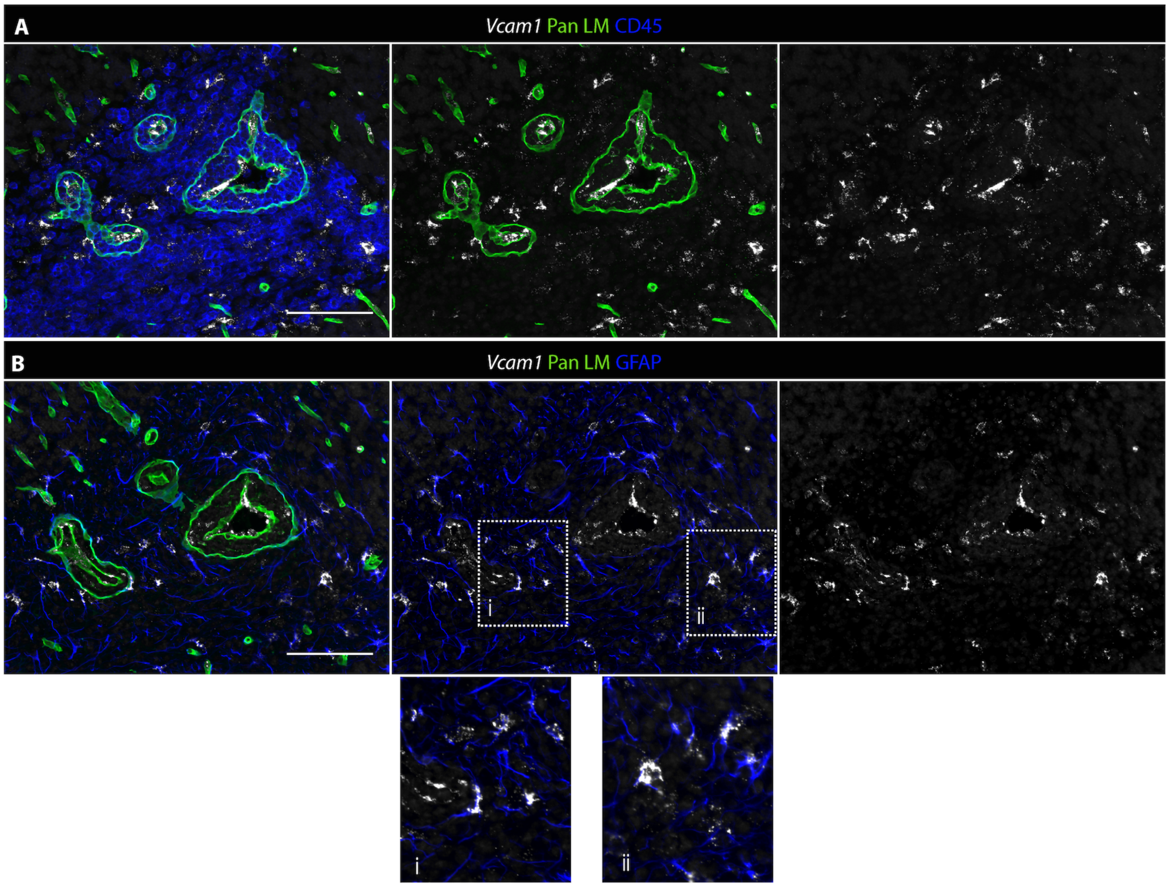

Fig. S11 RNAscope for *Vcam1* mRNA in EAE brain sections coupled with costaining for pan-laminin (Pan LM) to mark perivascular cuffs and either (A) CD45 to visualize the extent of leukocyte infiltration, or (B) GFAP to mark astrocytes. Boxed areas in B (i, ii) are shown at higher magnifications in the lower panels. Scale bars are 100 μm.

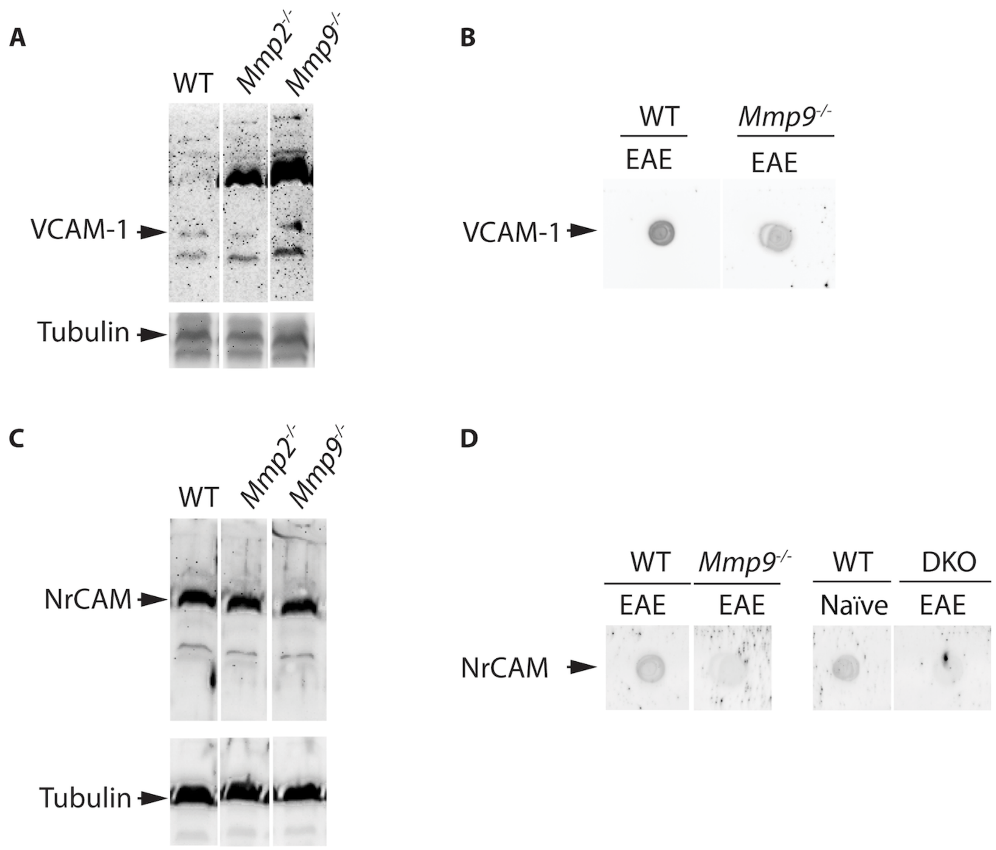

Fig. S12 Immunoblots of cerebellum extracts for full length NrCAM and VCAM-1 and CSF samples for soluble NrCAM and VCAM-1. (A) Western blots for VCAM-1 in cerebellum extracts from EAE WT, *Mmp2*$^{-/-}$ and *Mmp9*$^{-/-}$ samples and (B) dot blots for soluble VCAM-1 in EAE WT and *Mmp9*$^{-/-}$ CSF samples. (C) Western blots for NrCAM in cerebellum extracts from EAE WT, *Mmp2*$^{-/-}$ and *Mmp9*$^{-/-}$ samples and (D) dot blots for soluble NrCAM in EAE WT and *Mmp9*$^{-/-}$ CSF samples; and naïve WT and DKO samples. Data shown are exemplary of 3 different mice.

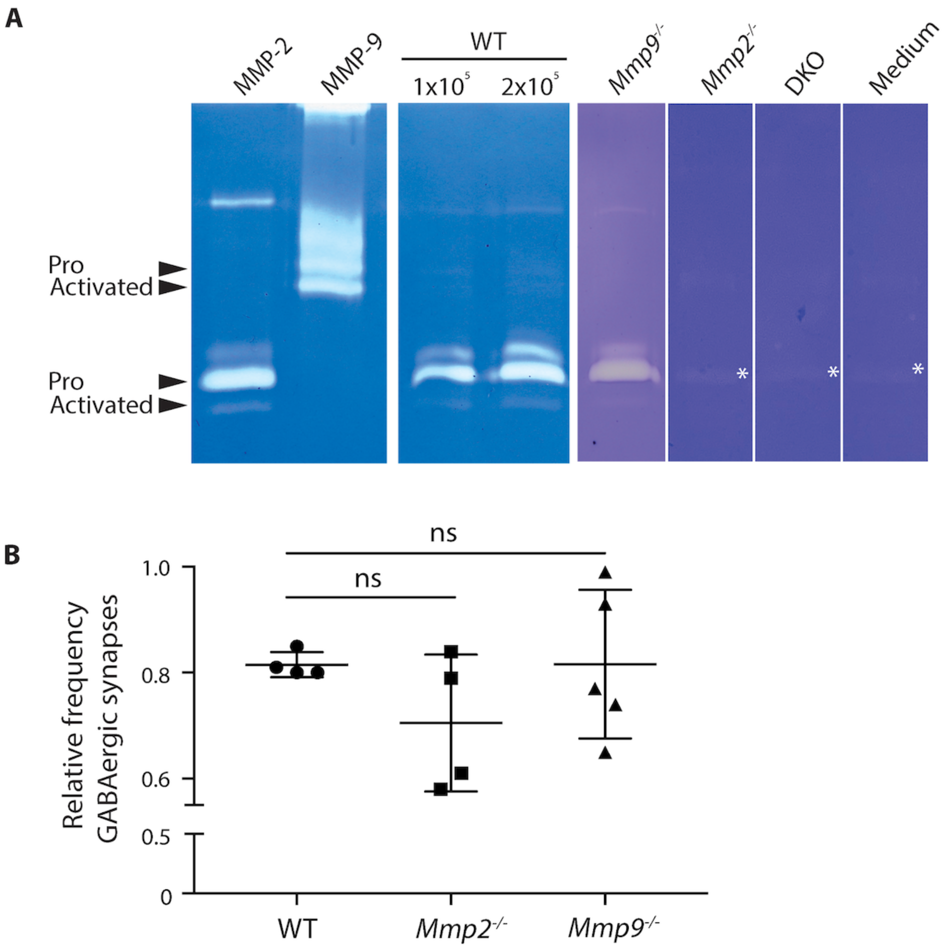

Fig. S13 Synapse development in WT, *Mmp2*$^{-/-}$ and *Mmp9*$^{-/-}$ astrocyte neuronal cocultures. (A) Gelatin gel zymography of conditioned media from WT, *Mmp2*$^{-/-}$ and *Mmp9*$^{-/-}$ and DKO astrocyte/neuronal coculture (approx. 1:1 ratio of astrocytes to neurons) or medium alone; $1x10^5$ and $2x10^5$ total number of cells (WT) were used in the coculture and reflect biological replicates; $2x10^5$ total number of cells were used for *Mmp2*$^{-/-}$, *Mmp9*$^{-/-}$ and DKO cultures; asterisk mark bands arising from low levels of serum contamination (<0.05%). (B) Relative abundance of glutamatergic and inhibitory GABAergic synapses in astrocyte neuronal cocultures. Statistical analysis of 4-5 experiments with separate culture preparations; data are expressed as relative frequency of GABAergic compared to glutamatergic synapses. Data are means ± SD with 2 replicates and 7-8 regions analysed/experiment. Statistical analysis was Student's T-test.

Table S1. Relapsing-remitting multiple sclerosis (RRMS) and control somatoform CSF samples tested

Samples	Diagnose	Treatment, Status	Gender, Age (years)
MS1	RRMS	Naïve, relapse	Female, 24
MS2	RRMS	Naïve, relapse	Female, 20
MS3	RRMS	Naïve, relapse	Female, 25
MS4	RRMS	Naïve, relapse	Male, 42
MS5	RRMS	Naïve, remission	Male, 19
MS6	RRMS	Naïve, relapse	Female, 35
MS7	RRMS	Naïve, relapse	Female, 30
MS8	RRMS	Naïve, relapse	Female, 57
MS9	RRMS	Naïve, relapse	Female, 28
MS10	RRMS	Naïve, relapse	Female, 28
MS11	RRMS	Naïve, relapse	Male, 45
MS12	RRMS	Naïve, relapse	Female, 48
MS13	RRMS	Naïve, relapse	Female, 28
MS14	RRMS	Naïve, relapse	Female, 58
MS15	RRMS	Naïve, relapse	Male, 69
MS16	RRMS	Naïve, relapse	Male, 44
C1	Somatoform	None	Female, 23
C2	Somatoform	None	Female, 23
C3	Somatoform	None	Female, 25
C4	Somatoform	None	Female, 32
C5	Somatoform	None	Male, 18
C6	Somatoform	None	Male, 23
C7	Somatoform	None	Female, 30
C8	Somatoform	None	Female, 57
C9	Somatoform	None	Female, 28
C10	Somatoform	None	Female, 28
C11	Somatoform	None	Male, 44
C12	Somatoform	None	Female, 49
C13	Somatoform	None	Female, 28
C14	Somatoform	None	Female, 57
C15	Somatoform	None	Male, 69
C16	Somatoform	None	Male, 44

Table S2. Antibodies employed in immunofluorescence staining, flow cytometry and Western blot analyses

Antigen	Antibody description/name/clone	Use	Reference/source
Pan-laminin	Rabbit anti-mouse laminin 111 (455)	IF	*(65)*
Laminin γ1	Rat anti-mouse laminin γ1 (3E10)	IF	*(65)*
GFAP	Cy3 mouse anti-pig GFAP, clone G-A-5<br>Alexa Fluor 488 mouse anti-pig GFAP, clone G-A-5<br>Guinea pig polyclonal anti-human GFAP	IF, FACS<br>IF<br>IF	Sigma, Germany, C9205<br>eBioscience, Germany, 53-9892<br>Synaptic Systems 173004
GLAST	APC mouse anti-human GLAST	FACS	Miltenyi Biotec, 130-123-641
VCAM-1	Rat anti-mouse VCAM-1 (M/K-2)<br>Rabbit anti-human VCAM-1	IF, WB, ELISA<br>IF	(*66*)<br>Sigma-Aldrich, HPA034796
CD11b	FITC rat anti-mouse CD11b	FACS	BD Pharmingen
CD45	Rat anti-mouse CD45.2 (30G12)	IF	BD Pharmingen
N-Cadherin	Rabbit anti-mouse N cadherin	IF	Abcam, ab76057
Cadherin-4	Sheep anti-mouse cadherin-4	IF	R&D Systems, AF6677
Cadherin-11	Rabbit anti-mouse cadherin-11<br>Rabbit anti-human OB cadherin	IF<br>IF	Invitrogen, 71-7600<br>Abcam, ab151302
NrCAM	Rabbit anti-mouse NrCAM<br>Mouse anti-human NrCAM	IF<br>WB	Abcam, ab24344<br>R&D Systems, MAB20341
Notch3	Goat anti-mouse Notch3	IF	R&D Systems, AF1308
ADAM10	Rabbit anti-human ADAM10 (C-terminal)<br>Rabbit anti-mouse ADAM10 (extra cellular)	IF, WB<br>IF, WB	Abcam, ab1997<br>Invitrogen, PA5-87899
Agrin	Rabbit anti-mouse full length agrin	IF	(*67*)
vGat	Rabbit anti-rat vGAT (GABA and glycine transporter)	IF	Synaptic Systems, 131002
vGlut1	Mouse anti-rat Glut1 Clone 317D5	IF	Synaptic Systems, 135311
MAP2	Mouse anti-human MAP2	IF	Synaptic Systems, 188011

Table S3. Primers used for PCR analysis

Gene name	Sequence (5` - 3`)	
ADAM10	Forward	GCAACATCTGGGGACAAACT
	Reverse	TTGCACTGGTCACTGTAGCC
Agrin	Forward	CACCGGGGACACTAGAATCTT
	Reverse	GAGCTACCATAGCAGGGCA
Cadherin 4	Forward	CAGGCCACTGACATGGAAGG
	Reverse	ATGATTCGGTAGACGGCGTTC
Gapdh	Forward	TGGCCTTCCGTGTTCCTAC
	Reverse	GAGTTGCTGTTGAAGTCGCA
N-cadherin	Forward	TTC AAG GTG GAC GAG GAC GG
	Reverse	TTG ATT GGC GGG ATG ACC CA
Cadherin 11	Forward	CTGGGTCTGGAACCAATTCTTT
	Reverse	GCCTGAGCCATCAGTGTGTA
Notch3	Forward	CGTGTGGCCTCTTTCTACTGT
	Reverse	GCACCAATCGAGCACTCATC
NrCAM	Forward	AACCTGAGTATGCAGTCGTCC
	Reverse	GTTGGAGTAGGAGCGACAACC
Vcam-1	Forward	AGTTGGGGATTCGGTTGTTCT
	Reverse	CCCCTCATTCCTTACCACCC
